# Transcriptional Response in Human Jurkat T Lymphocytes to a near Physiological Hypergravity Environment and to One Common in Routine Cell Culture Protocols

**DOI:** 10.3390/ijms24021351

**Published:** 2023-01-10

**Authors:** Christian Vahlensieck, Cora Sandra Thiel, Meret Mosimann, Timothy Bradley, Fabienne Caldana, Jennifer Polzer, Beatrice Astrid Lauber, Oliver Ullrich

**Affiliations:** 1 Institute of Anatomy, Faculty of Medicine, University of Zurich, Winterthurerstrasse 190, 8057 Zurich, Switzerland; 2 Department of Machine Design, Engineering Design and Product Development, Institute of Mechanical Engineering, Otto-von-Guericke-University Magdeburg, Universitätsplatz 2, 39106 Magdeburg, Germany; 3 Space Life Sciences Laboratory (SLSL), Kennedy Space Center, 505 Odyssey Way, Exploration Park, FL 32953, USA; 4 UZH Space Hub, Air Force Center, Air Base Dübendorf, Überlandstrasse 270, 8600 Dübendorf, Switzerland; 5 Ernst-Abbe-Hochschule (EAH) Jena, Department of Industrial Engineering, Carl-Zeiss-Promenade 2, 07745 Jena, Germany; 6 Zurich Center for Integrative Human Physiology (ZIHP), University of Zurich, Winterthurerstrasse 190, 8057 Zurich, Switzerland

**Keywords:** hypergravity, transcriptomics, mechanotransduction, lymphocytes, centrifugation

## Abstract

Cellular effects of hypergravity have been described in many studies. We investigated the transcriptional dynamics in Jurkat T cells between 20 s and 60 min of 9 g hypergravity and characterized a highly dynamic biphasic time course of gene expression response with a transition point between rapid adaptation and long-term response at approximately 7 min. Upregulated genes were shifted towards the center of the nuclei, whereby downregulated genes were shifted towards the periphery. Upregulated gene expression was mostly located on chromosomes 16–22. Protein-coding transcripts formed the majority with more than 90% of all differentially expressed genes and followed a continuous trend of downregulation, whereas retained introns demonstrated a biphasic time-course. The gene expression pattern of hypergravity response was not comparable with other stress factors such as oxidative stress, heat shock or inflammation. Furthermore, we tested a routine centrifugation protocol that is widely used to harvest cells for subsequent RNA analysis and detected a huge impact on the transcriptome compared to non-centrifuged samples, which did not return to baseline within 15 min. Thus, we recommend carefully studying the response of any cell types used for any experiments regarding the hypergravity time and levels applied during cell culture procedures and analysis.

## 1. Introduction

The gravity of Earth (g) as the acceleration force resulting from the mass of Earth and directed to the center of Earth’s mass with an approximate value of 9.81 m/s^2^ near Earth’s surface, is one of the fundamental conditions and determinants for all life on Earth.

Nevertheless, based on the constant gravitational acceleration on Earth, human cells and tissues are subjected to additional acceleration forces, on the one hand physiologically as in circulating cells in the pulsatile blood stream, or through the development and use of advanced technology as in aircrafts and spaceflight. Fighter pilots are exposed to temporary high hypergravity levels, with accelerations up to about 6 Gz during training and deployment, with peaks up to 9 Gz in high performance aircrafts [1]. Trained centrifuge participants have tolerated 3 Gx for 30 min [2], 8 Gx for 3 min [3], 12 Gx for 30 s, and 15 Gx for 10 s [4,5]. While accelerations on a suborbital spaceflight with Blue Origin’s New Shepard reached up to 30 s above 3 Gx and briefly up to 6 Gx on re-entry [6], the crew of the Space X Crew Dragon is exposed to up to 4.5 Gx for several minutes during second stage burn. Additionally, the use of hypergravity by human centrifugation is routine not only in research, testing and training of fighter pilots and astronauts, but also as a possible countermeasure to treat deconditioning after space flight. Interestingly, hypergravity protocols with therapeutic intent are also being used sporadically to treat various ground-based pathologies, such as peripheral obstructive arteriopathies, coronary artery disease, lymphedema and the Raynaud phenomenon [7]. Thus, exposure to a hypergravity environment occurs in many fields of human activity.

Even under physiological conditions on Earth, cells of the human body are regularly exposed to hypergravity. As an example, cells of the lymphatic system are characterized by enormous dynamics during circulation and recirculation between lymphoid tissues via the blood stream as a function of the adaptive immune system [8]. Lymphocytes circulating in the bloodstream receive pulsatile accelerations of about 5.5 m/s^2^ [9,10] up to 15 m/s^2^ [11]. With a transit time in the circulating blood stream of about 30 min [12,13], lymphocytes are thus subjected to recurrent accelerations over an extended period of time.

First experiments with isolated lymphocytes in altered gravity revealed not only the landmark discovery of strong cellular effects of microgravity [14], but also of hypergravity [15], including enhanced lymphocyte activation [16] and activation of metabolic pathways involved in cytokine secretion and increased expression of immunoregulatory molecule receptors [15]. Effects of hypergravity exposure have been described in many studies in vitro as well as in vivo [17], including in neutrophil granulocytes [18], melanocytes [19], fibroblasts [20], osteoblasts [21,22], chondrocytes [23], myoblasts [24,25], cardiomyocytes [26] and mesenchymal stem cells [27]. Most of these studies were performed in prolonged hypergravity exposure of at least hours and in hypergravity ranges mostly below 10 g [17]. Following the discovery of rapid cellular responses to hypergravity [28] and microgravity [29], we investigated the possibility of rapid transcriptome changes and discovered rapid changes after as little as 20 s of hypergravity or microgravity in human Jurkat T cells [30] and human myelomonocytic U937 cells [31] with subsequent adaptive responses in the minute range. The highly dynamic transcriptional response with rapid onset and rapid adaptation afterwards does not exclude, but rather suggests, that the relative difference and/or the speed of gravitational force alteration could be an important trigger for the cellular response (“reduced gravity paradigm” hypothesis according to van Loon) [32].

Follow-up studies demonstrated that a large fraction of transcripts altered in the acceleration phase of a suborbital ballistic rocket experiment could be re-detected by experiments with an experimental centrifuge on ground at 9 g [33]. Interestingly, and as a side result of these studies, we detected large differences in the transcriptome of cultivated Jurkat T cells before (cell culture controls) and after cell transfer into the experiment tubes (1 g controls) and found 2125 significantly upregulated and 496 significantly downregulated transcript clusters, solely due to the handling of the cells during experiment preparation [33].

This incidental observation led us to think more closely about potential effects of centrifugation methods commonly used in everyday laboratory work. This is because the majority of laboratory methods for harvesting and processing cells rely on centrifugation methods that commonly expose cells to a hypergravity environment of the order of 150 g to 300 g for 5 min [34,35]. After hypergravity exposure, living cells are subsequently cultured, used in experiments, or lysed or fixed for analysis. Considering the fact that hypergravity in the range of 1.8 g [30,31] or 9 g [33,36] caused fundamental structural [37], functional [38], and transcriptomic changes [30,31,33,36,39], the question immediately arises to what extent hypergravity-based laboratory methods influence the experiment or analysis results. Based on the high level of evidence that hypergravity has not only significant but also quantitatively meaningful effects on cells [17], it would potentially be useful to detect and quantify the effects of hypergravity-based laboratory methods using adequate control assays and/or internal controls. For this reason, our study also examined whole-genome gene expression in Jurkat T cells after various time points following application of a routine centrifugation protocol as widely used to harvest cells for subsequent RNA analysis.

In a previous RNA-Seq experiment with human Jurkat T cells, we already obtained gene expression data after two time points of 3 min and 15 min incubation in 9 g hypergravity [40]. The aim of this study was to capture transcriptional dynamics at much higher temporal resolution, to perform 3D gene distribution analysis, chromosomal distribution analysis, and transcript distribution analysis, and to make comparisons with results obtained on other platforms. In addition, we used rRNA-depletion libraries to be able to properly cover transcripts that did not finish transcription and therefore lack a poly-A-tail.

## 2. Results

**Figure 1:**
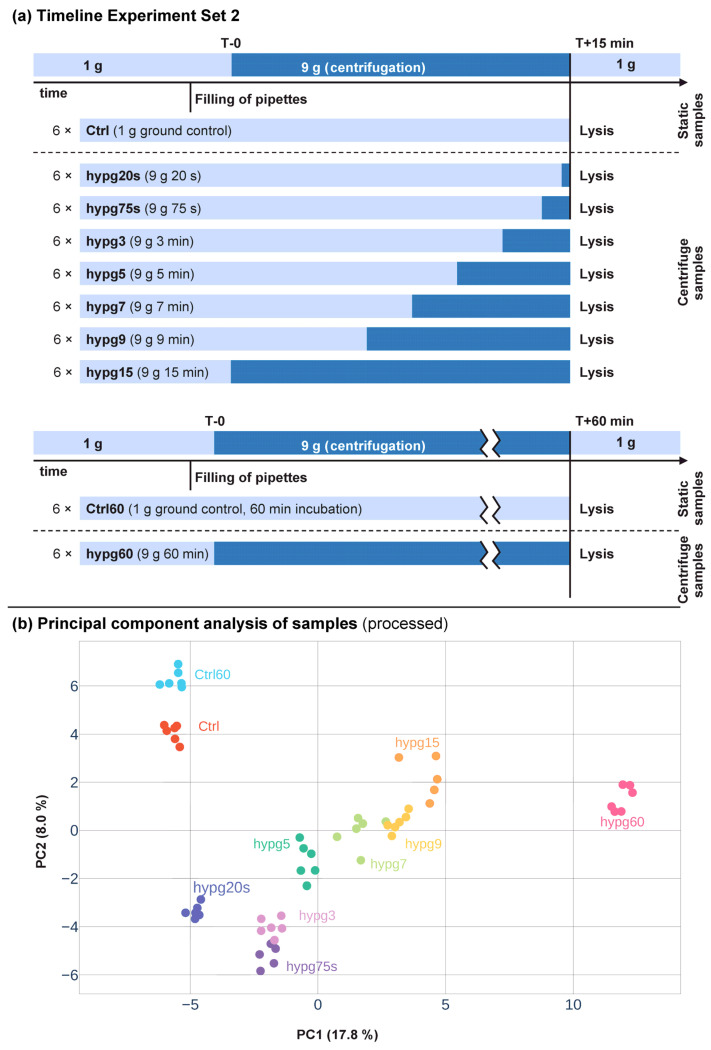
Experiment overview. (**a**) Experiment fixation scheme. The experimental conditions from which samples were acquired are displayed. There were two experimental sets, one with 15 min incubation time, one with 60 min incubation time. For the first set, Jurkat T cells were filled into 1 mL pipettes, incubated for 15 min and then rapidly emerged into RLT lysis buffer. The set contained 8 sample groups, with 6 samples per group each. Ctrl was incubated for 15 min at 1 g gravity, hypg20s was incubated for 14 min 40 s at 1 g gravity and then exposed to 9 g for 20 s on a pipette centrifuge before lysis (indicated in dark blue), hypg75s for 13 min 45 s at 1 g and consequently 75 s at 9 g, hypg3 for 12 min at 1 g and 3 min at 9 g, hypg5 for 10 min at 1 g and 5 min at 9 g, hypg7 for 8 min at 1 g and 7 min at 9 g, hypg9 for 6 min at 1 g and 9 min at 9 g, and hypg15 for 15 min at 9 g. The second set contained the groups Ctrl60 that incubated at 1 g for 60 min and hypg60 that was continuously exposed to 9 g for 60 min. From Figure 2 on, comparisons between the hypg condition and Ctrl, resp. between hypg60 and Ctrl60, are named hypg20 s and so on. (**b**) Principal component analysis of gene counts for all samples. Counts were processed by surrogate variable analysis batch effect removal to exclude sequencing-specific noise and to promote clustering of sample group. Percent of explained variance is given per axis.

The aim of this study was to assess the short-term effects of hypergravity on the transcriptome of human Jurkat T cells with a high temporal resolution. In a previous study, we already investigated the effects of hypergravity after 3 min and 15 min of centrifugation (Experiment Set 1). We now wanted to analyze the short term effects in more detail and therefore tested eight centrifugation times in the range of 20 s up to 60 min (Experiment Set 2) (Figure 1a). All sample groups between 20 s up to 15 min of centrifugation were compared to control samples incubated for 15 min without centrifugation. For the samples that were centrifuged for 60 min, a separate control group was incubated for 60 min.

The shortest hypergravity exposure, hypg20s (20 s), was chosen based on the duration of the hypergravity phase on previously performed parabolic flights (compare [30,41]).The hypg75s (75 s) group represented the acceleration phase of previous ballistic rocket studies [30,41]. We also repeated two time points (hypg3 (3 min) and hypg15 (15 min)) from one of our previous studies named here Experiment Set 1, [40]. There, we could show an inversion point of differential gene expression at or after 5 min of hypergravity, which we described as transcriptional rebound effect [40]. Based on these results, we added the time points hypg5 (5 min), hypg7 (7 min), and hypg9 (9 min), to cover a potential inversion point. In contrast to the study represented by Experiment Set 1, where used poly-A libraries for RNA-Seq, we used rRNA-depletion libraries in this study (Experiment Set 2) to be able to also properly cover unfinished transcripts lacking a poly-A-tail.

For a first impression of the expression differences between the sample groups, we performed a principal component analysis (Figure 1b). We made a svaseq batch effect correction to compensate for sequencing artifacts. Interestingly, the principal component analysis showed a clear clustering of the individual sample groups with a temporal sequence associated with the duration of centrifugation indicated by a grey bar in Figure 1b.

We further analyzed the RNA-Seq data sets on a general level (i.e., all transcript types together, called “the regular transcriptome level”), as well as separately on the spliced and unspliced transcriptome level, as previously described by our group [40].

**Figure 2:**
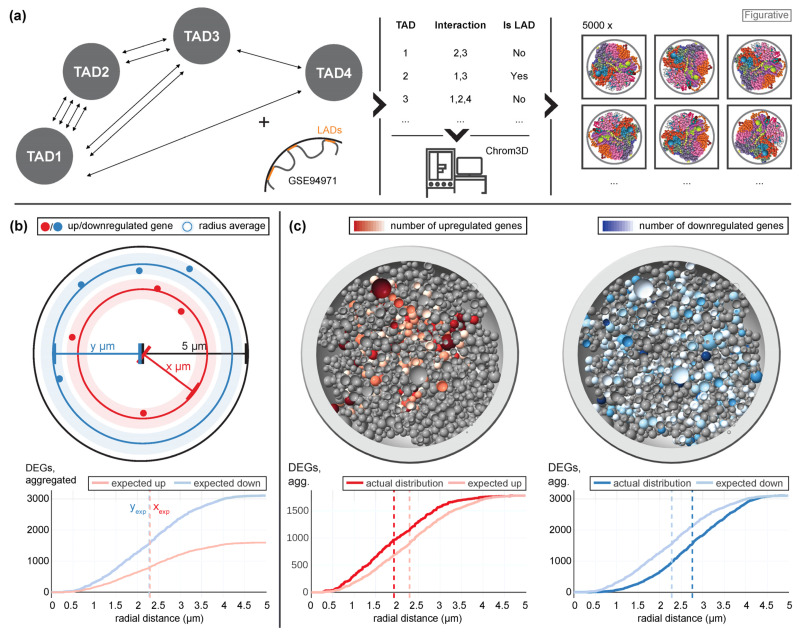
Conceptualization of 3D gene distribution analysis. (**a**) Hi-C sequencing data from the 4th Swiss Parabolic Flight Campaign of Jurkat T cells were used to determine which topology-associated domains (TADs) interacted with each other. Additionally, lamina-associated domains of Jurkat T cells were utilized from the external GSE94971 data set. These two data sets were converted into a table, listing per TAD significantly interacting other TADs (implying proximity) and if this TAD is a LAD (increasing the probability to localize at the more remote parts of the nucleus). From this, the bioinformatic package Chrom3D calculated a set of 5000 potential 3D bead structures of the chromatin that fulfill the conditions from the table. These beads corresponded to TADs or continuous regions without TADs and could carry several genes. Integrity tests on the models can be found in Supplementary Figure S1. (**b**) Example of a 3D gene distribution analysis. For each gene, the radial distance to the nuclear center of the bead where the gene is located was determined. The expected distribution of differentially upregulated and downregulated genes (random drawing of genes from the set of all genes) and the actual distribution was analyzed. The average radius was calculated for the expected and the actual distribution of differentially upregulated/downregulated genes. In the diagram, the expected distributions of upregulated and downregulated genes are shown exemplary for the data set hypg15 of Experiment Set 1. The plot shows the number of differentially expressed genes (DEGs) that had a radial distance smaller or equal to the distance on the horizontal axis. The average distances (dashed lines) were the same for both expected distributions. (**c**) The actual distributions of significantly upregulated and downregulated genes are shown for the same data set as in (**b**). One example model of the generated 5000 models is displayed, the number of significantly upregulated or downregulated genes per bead is indicated by the color scheme. In the diagrams, the average distances of the actual distributions were shifted towards the expected average distances.

Our current model of how Jurkat T cells could respond to altered gravity included direct mechano-genetical coupling of gravitational forces acting on the cellular membrane to the nucleus, where genes are differentially expressed, depending on their nuclear location, due to conformational changes [39]. We wanted to assess if the differential expression pattern analyzed in this study supports our hypothesis. We therefore utilized a Hi-C data set from an experiment performed on the 4th Swiss Parabolic Flight Campaign (4SPFC) [39] and created a set of 3D models exploiting the Chrom3D pipeline [42] (Figure 2a). In detail, identified topology associated domains (TADs) were probed for significant interaction and then labelled as neighboring. Additionally, an external reference data set (GSE94971) [43] which contained lamina associated domains (LADs) of Jurkat T cells was used to label TADs as lamina associated. The nuclear radius was set at 5 µm in accordance with microscopy results [44]. The resulting table was then processed by Chrom3D, which generated a set of 5000 structural 3D models that all fulfilled the arrangement conditions derived from the table. The generated models could be validated successfully (Supplementary Figure S1).

Next, all genes were mapped to their corresponding beads in each model, either representing a TAD or the region between two TADs (Figure 2b). For significantly upregulated or downregulated genes, the radial distance to the center of the nucleus was calculated. First, the expected distribution under the null hypothesis was determined, which represented randomly drawing genes as differentially expressed from all genes. For this case, the average of the radial distribution was the same for upregulated (xexp) and downregulated (yexp) genes. In a next step, the actual distribution based on the real distribution of differentially up- or downregulated genes was calculated (Figure 2c), exemplary shown for one of the 5000 models. Here, genes that were upregulated tended to localize closer to the nuclear center than expected, and downregulated genes closer to the periphery than expected. In the analysis, this was consecutively calculated for all 5000 models, compared, and averaged.

**Figure 3:**
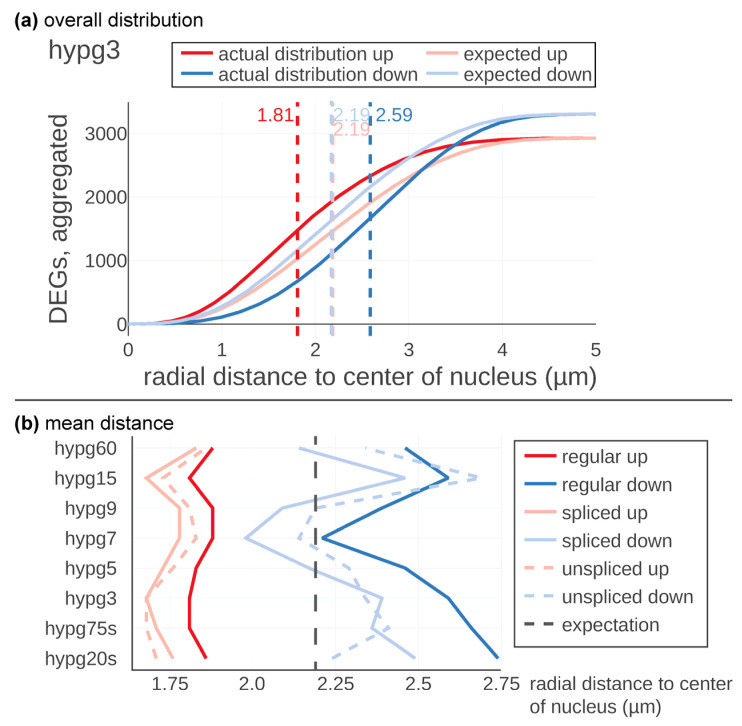
3D gene distribution analysis of Experiment Set 2 data. (**a**) Detailed analysis for one comparison, hypg3. The expected average distance for both significantly upregulated and downregulated genes was 2.19 µm from the center of the nucleus. Upregulated genes tended to localize more in the center with an average of 1.81 µm, downregulated more remote with an average of 2.59 µm. (**b**) Average distance of significantly up- and downregulated genes for all comparisons, for the standard differential expression analysis (regular), and displayed separately for the spliced and unspliced transcriptome.

Next, this method was applied to the Experiment Set 2 data and the actual and the expected distribution were compared (Figure 3). In Figure 3a, the results are displayed in detail exemplary for the time point hypg3. The expected average distance of up- and downregulated genes was located at 2.19 µm with respect to the center of the nucleus. The maxima of the two expectation curves did not co-localize since more genes were downregulated than upregulated at hypg3. The actual distribution of upregulated genes was shifted towards the center, at 1.81 µm distance, therefore having a difference of −0.38 µm. Downregulation was shifted to the periphery, at 2.59 µm on average and therefore shifted by +0.40 µm.

These calculations were performed for all eight time points for the regular, the spliced, and the unspliced transcriptome (Figure 3b). Downregulated genes showed a strong shift in radial average over time. After 20 s of hypergravity, the average distance with respect to the center of the nucleus was shifted to the periphery (2.75 µm). Then, the average distance was constantly decreased until 7 min of hypergravity exposure (hypg7) where it reached with 2.24 µm almost the expected calculated distance (2.19 µm). The average of the distance was thereafter significantly shifted again towards the periphery (hypg9, hypg15 and hypg60).

The spliced and unspliced tracks showed a similar behavior; however, the unspliced transcriptome showed after 20 s hypergravity exposure of almost the predicted value. The spliced transcriptome average for the downregulated transcripts was even further shifted towards the center of the nucleus than the curve representing the regular transcriptome (blue curves in Figure 3b). The upregulated genes also followed a temporal course but with a less pronounced magnitude (Figure 3b, red curves). Initially, genes were shifted inwards at 1.86 µm after 20 s of hypergravity. At hypg75s and hypg3, a maximum of 1.81µm was reached, then the average radial distance shifted more towards the predicted value. hypg7 and hypg9 showed minima values at 1.88 µm, then at hypg15 values were at 1.81 µm, while for hypg60 they moved back to 1.88 µm. The spliced and unspliced tracks developed in a similar fashion.

Downregulated genes appeared to dislocate towards the nuclear periphery with an intermediate return to baseline after 7 min of hypergravity exposure while upregulated genes were located more towards the center of the nucleus (Figure 3b).

**Figure 4:**
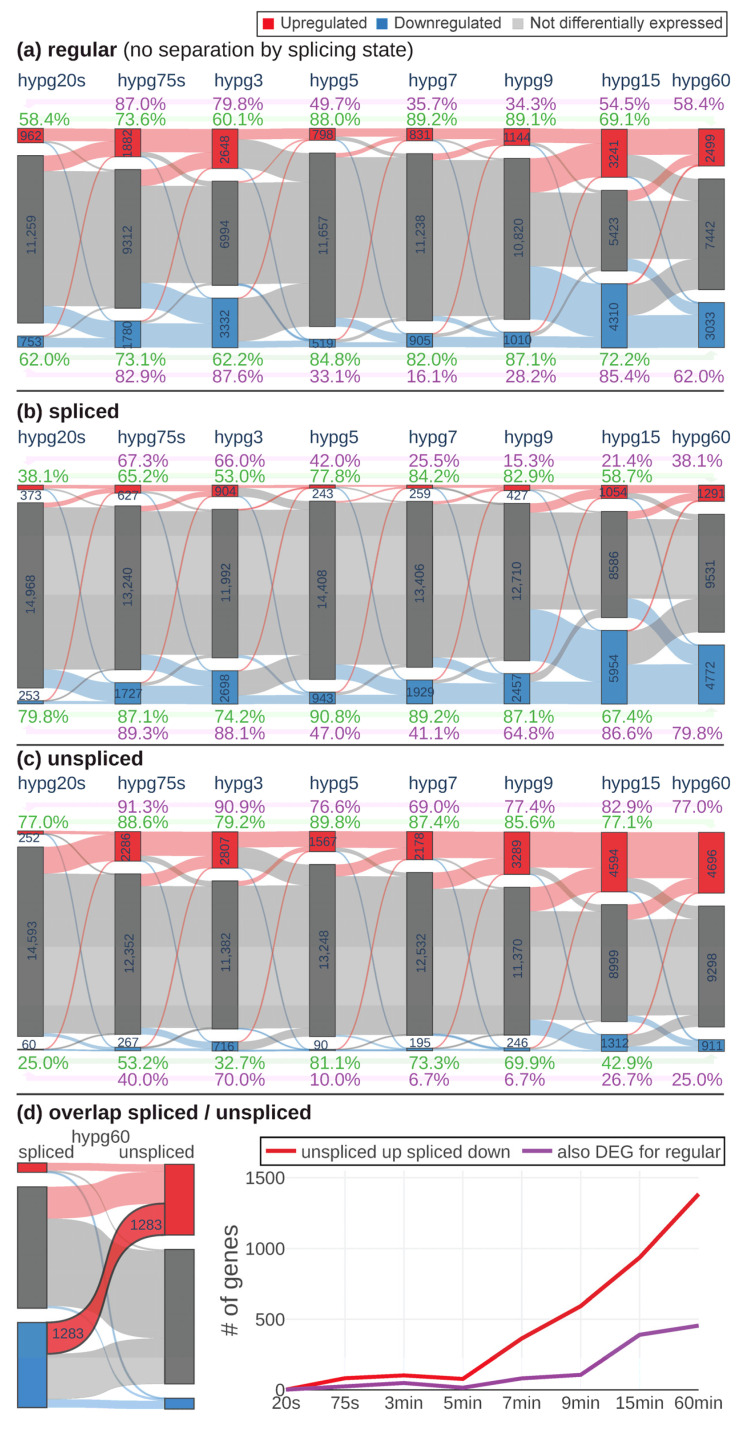
Time course of differential expression. For each comparison, the number of significantly upregulated, downregulated and not differentially expressed genes is given. Flow bars between the comparisons indicate how many genes were regulated in a certain direction in the following comparison. Percent values indicate the coherence of differential gene expression in relation to hypg20s (purple) and hypg60 (green), separately for upregulated (above diagram) and downregulated (below diagram) genes: When comparing DEGs to the DEGs of the reference, it was calculated how many of the genes of the smaller group were differentially expressed in the same direction of the larger group. The overall analysis was performed on the regular transcriptome (**a**), the spliced transcriptome (**b**), and the unspliced transcriptome (**c**). (**d**) Some genes were significantly downregulated in the spliced transcriptome and upregulated in the unspliced, exemplary shown for hypg60. Not all of them were differentially expressed in the regular transcriptome. The number of “unspliced up spliced down” genes is displayed per comparison. Additionally, the fraction of these genes that were also differentially expressed for the regular transcriptome is displayed.

To have a better understanding of the observed behavior of 3D distribution, the continuity of differential gene expression was probed by a Sankey diagram analysis (Figure 4a–c). For all comparisons, the number of significantly upregulated, downregulated, and not differentially expressed genes was determined (defined as FDR-adjusted *p* value < 0.05, no fold change cutoff). The behavior of genes from one group (e.g., upregulated) at the next comparison was indicated by connecting bands that showed how many genes were still upregulated, how many downregulated and how many were not differentially expressed any more. For the regular transcriptome (Figure 4a), the number of genes that were differentially expressed increased from hypg20s over hypg75s up to hypg3 with a peak of 2648 upregulated and 3332 downregulated genes. Then, it massively dropped at hypg5 and hypg7, only slightly increased at hypg9 and then affected most genes at hypg15 and partly still at hypg60. Genes had a tendency towards downregulation at the spliced transcriptome level (Figure 4b) and towards upregulation at the unspliced transcriptome level (Figure 4c). The behavior of a decreased effect strength between 5 and 7 min was also present on these two levels; however, hypg9 seemed to be already fully recovered on the unspliced level, since the number of DEGs was larger than at hypg3. The coherence of the gene expression response was tested by calculating the ratio of genes regulated in the same direction between two conditions compared to the size of the smaller of the two groups compared. This was done based on hypg20s (purple) and on hypg60 (green). Coherence of upregulated genes towards hypg20s was especially low for hypg7 and hypg9, where values for the regular track fell down to 34.3% and even to 15.3% for the spliced track (however, spliced upregulated genes were generally rare). They did not fully recover for hypg60. For upregulated genes, coherence towards hypg60 (in green) was especially high for hypg7 and hypg9, reaching values of up to 89.2% for the regular track. For downregulated genes, the situation was comparable, the coherence to hypg20s even dropped down to 16.1% at hypg7, which was unexpectedly low compared to hypg5 and hypg9 with roughly the same number of differentially expressed genes.

When comparing spliced and unspliced genes, it came to our attention that there were genes that were upregulated on the unspliced level and downregulated on the spliced level (Figure 4d). This was especially prominent for hypg60 (left side of the figure). When plotting the number of genes that showed this behavior, a continuous increase after starting at hypg7 emerged. However, only a fraction was parallelly differentially expressed on the regular transcriptome level. Interestingly, almost no genes showed the effect for hypg3, which had more overall DEGs than hypg5, hypg7, and hypg9.

Generally, the data showed a biphasic pattern with a peak of differentially expressed genes at hypg3 and a high coherence between comparisons, and almost no “unspliced up spliced down”-effect. After a transition period between hypg5, hypg7, and partly hypg9 (at least for the regular transcriptome), a second peak of differentially expressed genes was observed at hypg15. Coherence to the early comparisons was partly lost but was high within these early comparisons. Suddenly, many genes show an “unspliced up and spliced down”-response.

**Figure 5:**
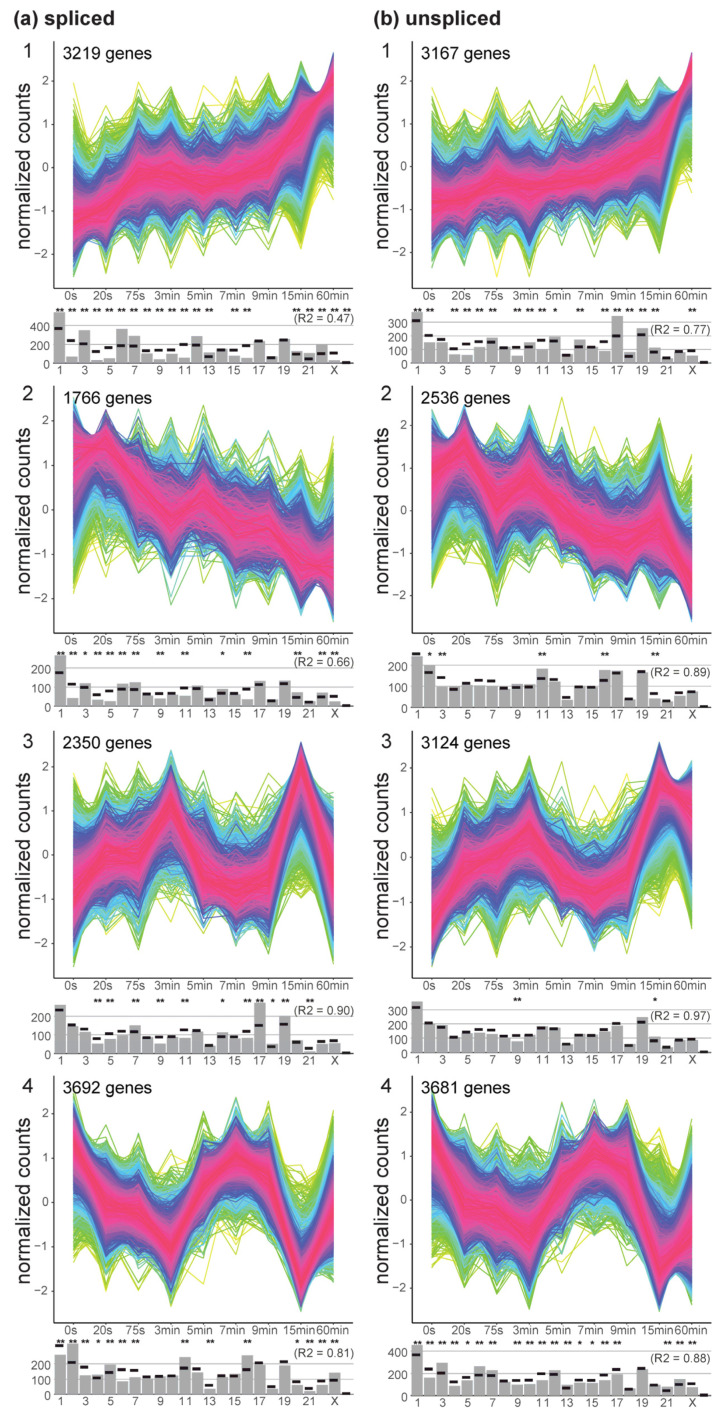
Temporal analysis with time course tool TCseq. Significantly changing normalized gene count courses were clustered into groups. Normalized counts were plotted against all sample groups, ordered by their length. Four groups were selected for the analysis as the best compromise between complexity and homogeneity of groups. For each group (1–4), the density of genes is displayed by a rainbow pattern, with purple/red representing most time courses. The distribution over the chromosomes is given below each group, actual value as a grey bar, expectation by random drawing as black dash. Significant differences (adjusted *p* < 0.01/0.05) in localization are indicated by */**. The correlation coefficient between the expected and the actual number of genes per chromosome was given for each diagram, the smaller the number the larger the deviations from a random distribution. The analysis was separately performed for the spliced (**a**) and unspliced (**b**) transcriptome. Genes that are in time course group 1 for (**a**) are not necessarily in group 2 for (**b**).

To have a better understanding of the temporal effects of hypergravity exposure with respect to differential gene expression response, a temporal clustering algorithm, TCseq [45], was applied on the normalized count data for the spliced and unspliced transcriptome (Figure 5). Four cluster groups gave the best compromise between compactness of the time course distributions and categorial differences between groups. For both the spliced and the unspliced data, one group with continuously increasing counts was identified (group 1). Transcripts in group number 2 showed a continuously decrease in gene expression. Group 3 displayed two peaks in differential gene expression, the first after 3 min and the second after 15 min. The opposite response could be observed for group 4 for both spliced and unspliced transcripts, with an initial loss until 3 min, recovery until 7 min, strong loss at 15 min, and at least for the spliced counts partial recovery until 60 min.

These four groups all carried between 1766 up to 3692 genes that were identified to have significantly altered counts over the time course. The distribution of these groups along the chromosomes varied greatly. On the spliced level, groups 1 and 2 deviated strongly from a random distribution over all chromosomes (R2 of 0.47 and 0.66), with a strong underrepresentation of chr2, groups 3 and 4 were more evenly distributed, however with a strong overrepresentation of chr17 for group 3. Unspliced transcripts in group 1 deviated greatly from a random distribution (R2 = 0.77), groups 2, 3, and 4 were almost evenly distributed. No significant shifts in gene biotype distribution between clusters had been identified (not displayed in Figure 5).

**Figure 6:**
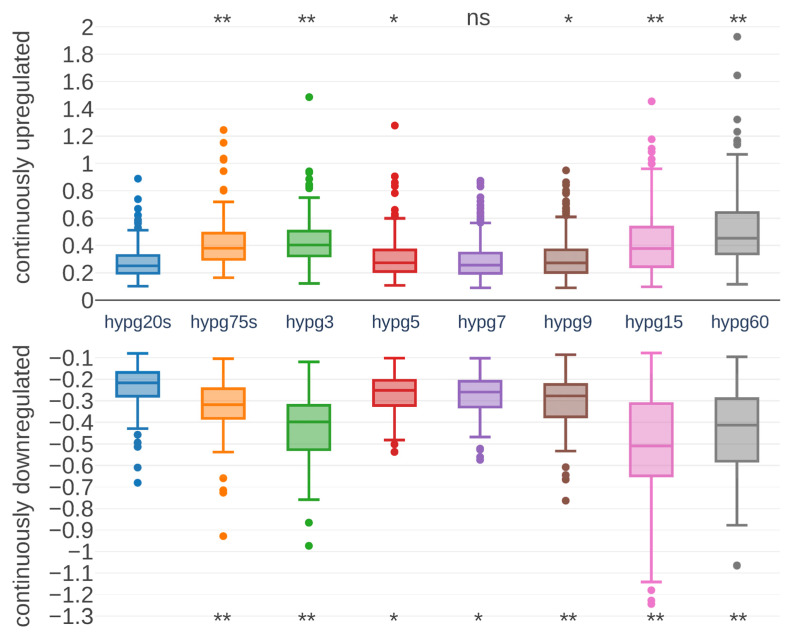
Distribution of fold changes for continuously differentially expressed genes. Genes that had an adjusted *p* value below 0.05 for all comparisons and always showed a positive (upper part of the diagram) or negative (lower part) fold change were selected. The distribution of fold changes on the vertical axis is plotted as a boxplot for each comparison on the horizontal axis. Statistical differences between the hypg20s and later comparisons have been assessed by a *t* test. Significance levels, based on FDR-corrected *p* values, are indicated above/below each comparison (*: *p*-value below 0.05, **: *p*-value below 0.01).

Next, we asked if also the strength of differential expression was affected. Therefore, we focused on genes that were permanently upregulated or downregulated at all comparisons. In total, 214 upregulated and 87 downregulated genes fulfilled these conditions. Their fold changes were tracked over time (Figure 6). Surprisingly, both permanently upregulated and downregulated genes had a peak in fold change at hypg3, then effect strength dropped with a minimum at hypg7 and began to rise again. However, hypg5 had a stronger median fold change than hypg20s, in contrast to the number of differentially expressed genes in Figure 4. For the upregulated genes, hypg60 had the strongest overall fold changes, while for the downregulated genes hypg15 had a stronger median but also a larger distribution. Therefore, not only the number of differentially expressed genes was affected over time but also the strength of the differential expression itself.

**Figure 7:**
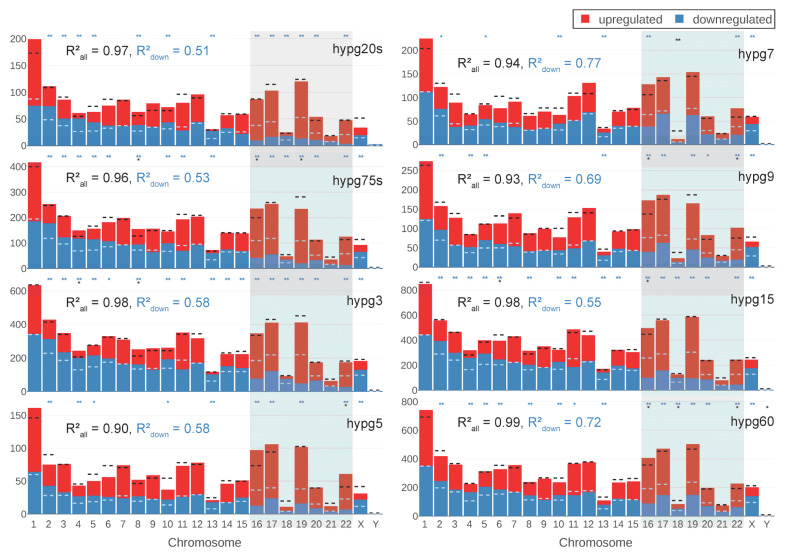
Chromosomal distribution of differential gene expression. Number of differentially upregulated (red) and downregulated (blue) genes per chromosome (horizontal axis) for all comparisons. The expected number of total DEGs per chromosome (based on the fraction of differentially expressed genes for all genes and the number of detected genes per chromosome and assuming a uniform probability of differential expression) is shown as a black dashed line, the expected number of downregulated genes out of up- and downregulated genes is shown as dashed light blue line. If the difference between expectation and actual number of genes was so large that it could not be explained from random distribution, this was indicated above (* for adjusted *p* values < 0.05, ** for below < 0.02), both for absolute number of genes and distribution between up- and downregulation. The correlation coefficient between the expected and the actual number of total DEGs/upregulated genes was given. The closer the correlation coefficient comes to 1, the more the actual number corresponds to the expected number.

Based on the 3D analysis from Figure 3 and the results from the study with Experiment Set 1, the chromosomal distribution of differentially expressed genes was analyzed. For each chromosome, the number of total DEGs and of upregulated versus downregulated genes was compared to the predicted value based on random drawing (Figure 7). If the differences were significantly larger (FDR-adjusted *p* value below 0.05/0.01), this was indicated. When comparing chromosomes, the deviations from expectation on the level of total number of differentially expressed genes were smaller than on the level of skew between up- versus downregulation. The small chromosomes chr16-chr22 (right side of the diagrams in Figure 7) showed an especially large tendency towards upregulation for hypg20s, hypg75s, hypg3, and hypg5. For hypg7 and hypg9, this effect was no longer given. The overall skew in up- versus downregulation was indicated by the Spearman correlation coefficient between actual number of genes versus prediction. It was especially skewed for hypg20s-hypg5 (much smaller than 1), and became less pronounced for hypg7 with 0.77 up to 0.72 for hypg60, with hypg15 as exception. This corresponds to the results from Figure 3, where hypg7 and hypg9 did not show a radial distribution behavior as pronounced as the other time points. However, the skewness of overall number of DEGs increased for hypg5 with 0.90, until hypg9 with 0.93 compared to almost perfect correlation for all other time points. This was a puzzling perspective on the three comparisons hypg5, hypg7, and hypg9, which separated the early from the late time points in Figure 4.

**Figure 8:**
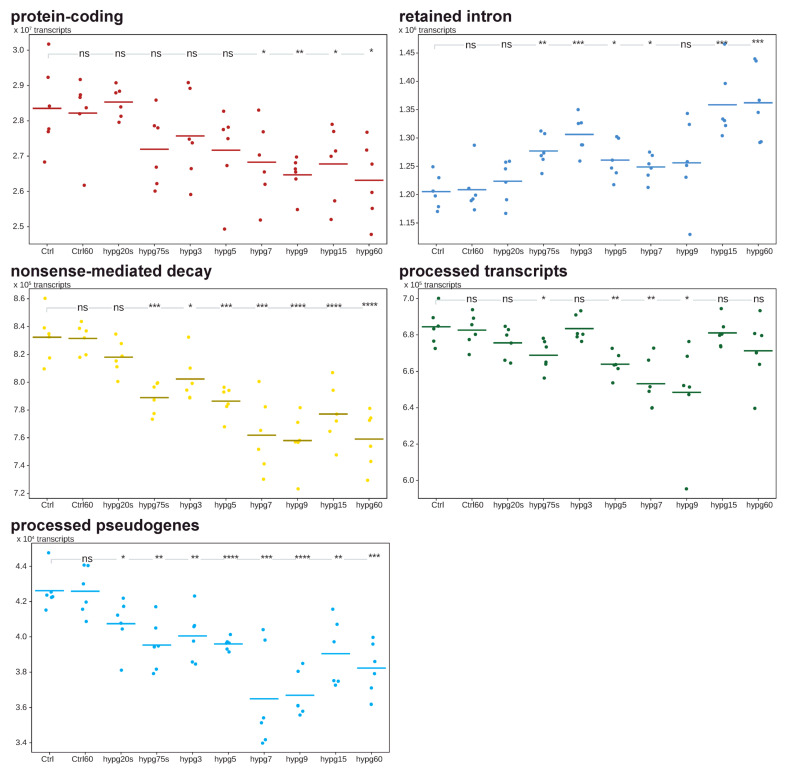
Transcript distribution analysis. The data set was analyzed on the level of transcripts. The aggregated counts of the five most represented biotypes, protein-coding, retained intron, nonsense-mediated decay, processed transcripts (usually lacking an open reading frame) and processed pseudogenes (usually lacking an open reading frame) are shown. The sum of counts is shown for each sample, the averages of sample groups are indicated by dashes. In the top of each diagram, it is indicated if the differences between Ctrl and any other group is significant (* for adjusted *p* < 0.05, ** for adjusted *p* < 0.01, *** for adjusted *p* < 0.001, **** for adjusted *p* < 0.0001, based on Student’s *t* test) or not (ns).

We further analyzed the differentially expressed transcripts and allocated them to the five most prominent biotypes, protein-coding, retained intron, nonsense-mediated decay, processed transcripts and processed pseudogenes (Figure 8). The biotype is important for the biological role of a transcript. Protein-coding transcripts are capable of being read by ribosomes at mostly high efficiency. Retained intron transcripts can still be translated, however often at reduced efficiency and are prone to degradation, nonsense-mediated decay tends to quick degradation, and processed transcripts and processed pseudogenes usually stem from reversely transcribed RNA that has been reinserted into DNA and are mostly unable to be translated anymore [46]. The sum of these transcripts per sample was compared between groups and significant differences indicated.

Protein-coding transcripts formed the majority with 90.4% of all counts. After hypg20s, the average constantly declined, and became significant for all comparisons starting from hypg7. The number of transcripts assigned to retained introns, the second-largest transcripts group, increased significantly up to hypg3, then slightly decreased, until emerging again for hypg15 and hypg60. Nonsense-mediated decay transcripts showed significantly reduced counts for all comparisons except for hypg20s and progressively became less until hypg9. The amount of processed transcripts significantly fell until hypg9 and thereafter returned to normal levels. In the case of processed pseudogenes, the amount of transcripts was significantly decreased for all time points of hypergravity exposure.

A trend towards intron retention became evident from these data. Whilst protein-coding genes and partly also nonsense-mediated decay followed a continuous trend of downregulation, intron retention would match the biphasic behavior described above. The two pseudogene species showed a transient behavior that would match the time courses 3 and 4 from Figure 5.

**Figure 9:**
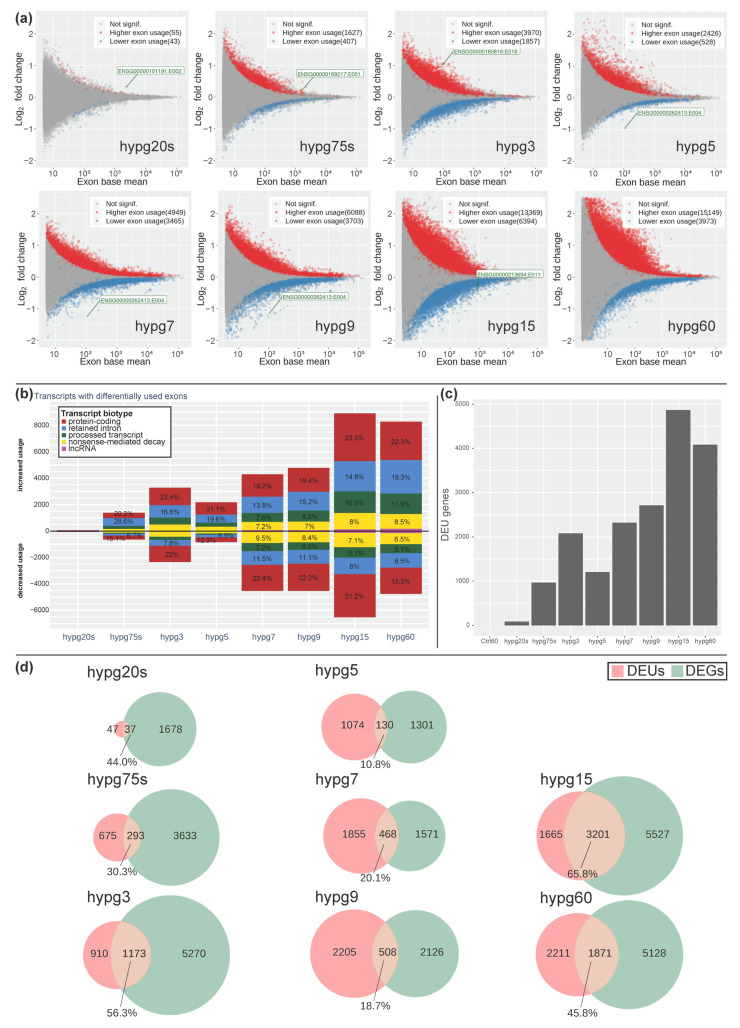
Differential exon usage/splicing dynamics. (**a**) MA plot for fold changes of exons for all comparisons in time. Exons that showed significantly increased (red) or decreased (blue) usage are highlighted.. The exon with the lowest *p* value is highlighted for each comparison. (**b**) The associated transcripts of differential exon usage events. Significantly increased exon usage is given above the zero line, decreased below. The transcripts were categorized by transcript biotype. Alterations in protein-coding exons still leading to translatable transcripts; transcripts with exons flagged as retained introns, processed transcripts and nonsense-mediated decay lead to transcripts that are not translated into proteins or show decreased translation rates and are likely prone to early degradation. (**c**) The number of associated genes with exons that were affected with significant differential exon usage for all comparisons. (**d**) Overlap between genes with differential exon usage (DEU, red) with genes that were differentially expressed (DEG, green). Fraction of overlapping DEU compared to all DEU is indicated in percent. An analysis with DEGs for the spliced and the unspliced transcriptome can be found in Supplementary Figure S2.

The significant alterations in transcript type distribution over time let us wonder if there could also be noticeable effects on the level of exons. We performed a DEXSeq analysis and scrutinized differential exon usage (Figure 9). The number of significantly higher or lower exon usage per condition was determined with the help of an MA plot (Figure 9a). For all hypergravity exposure times, differential exon usage (DEU) could be detected. In case of increase exon usage, the values constantly increased over time with an exception for the time point hypg5. In case of exons showing a decreased usage, we observed a similar trend, i.e., the number of exons with decreased usage was rising with an exception for time point hypg5 and additionally hypg60 (Figure 9a). The drop in DEUs at hypg5 was compensated more rapidly than for differential gene expression (Figure 4), at hypg7 already a new maximum was reached. This also holds true for the affected transcripts (Figure 9b) and the corresponding number of genes with DEU (Figure 9c). Furthermore, the distribution of transcripts with differential exon usage among the different biotypes was non-even (Figure 9b). Protein-coding transcripts appeared relatively balanced in terms of increased to decreased usage; however, retained intron transcripts had a strong shift towards increased usage. This partially overlapped with the general trend in transcript type counts shown in Figure 8.

When genes with differential exon usage were overlapping with genes with differential gene expression, a highly dynamic relation emerged (Figure 9d). Initially, the number of DEUs was small compared to DEG. At hypg5, they were almost equal (1204 DEUs, 1431 DEGs), and for hypg7 and hypg9 the number of DEUs even surpassed the number of DEGs. The number of genes that were both DEUs and DEGs varied greatly over time. It rose to 56.3% at hypg3, then dropped to only 10.8% at hypg5, stayed low until hypg9, and then rose to 65.8% for hypg15. The same analysis for differential gene expression for the spliced and unspliced transcriptome can be found in Supplementary Figure S2. The distribution of DEU genes along the chromosomes can be found in Supplementary Figure S3. Genes affected by DEU were not automatically DEGs and could stay a significant fraction on their own, also showing a biphasic course.

**Figure 10:**
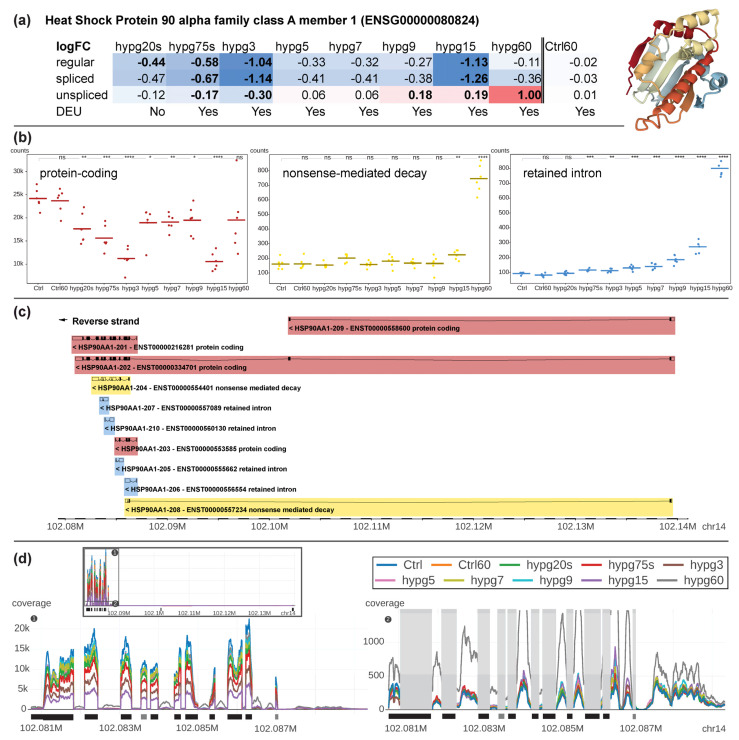
Detailed analysis of *HSP90AA1*, the gene with multiple transcript isoforms that showed the strongest significantly differential expression while having more than 1000 counts in each condition. (**a**) Fold changes for each comparison for the regular, spliced and unspliced transcriptome. The fold change is continuously color-coded, with blue for downregulation, white for no differential expression and red for upregulation. Significant differential expression is indicated by bold font. The protein is displayed, based on PDB structure 1BYQ. (**b**) Transcript counts aggregated by transcript biotype per comparison. Each sample is represented by one point, sample group averages by dashes. Protein-coding contains the summarized counts of transcript variants ENST00000216281, ENST00000334701, ENST00000553585, and ENST00000558600, nonsense-mediated decay of ENST00000554401, and ENST00000557234, and retained intron of ENST00000557089, ENST00000560130, ENST00000555662, and ENST00000556554. (**c**) Distribution and structure of all transcripts of HSP90AA1. The gene is on the reverse strand; therefore, first exons are located on the right. (**d**) Sequencing coverage along the gene on chromosome 14. The upper box shows the entire gene, sub-plots 1 and 2 focus on the first part of the gene that carries most reads. Sub-plot 1 focusses on exonic regions. Sub-plot 2 hides the strongly covered exons (grey boxes) and focusses on the intronic regions. Coverage for different points in time is shown by different colors. Exon location is indicated by black boxes (main exons), the first grey exon corresponds to a nonsense-mediated decay transcript, the second to a retained intron transcript.

To better understand the implications of the effects described above, a bona fide example of a gene that was affected both by differential gene expression and exon usage was selected. Additionally, this gene should have more than one transcript isoform from more than one transcript biotype and should have a substantial number of counts to be robustly identified. *HSP90A1*, a gene coding for an inducible molecular chaperone that aids in protein folding using an ATPase activity, fulfilled all requirements (Figure 10). It is localized on chr14 and has a length of 58,958 bp. The gene was significantly downregulated from hypg20s to hypg3 for the regular transcriptome, and also downregulated for both the spliced and unspliced transcriptome for hypg75s and hypg3 (Figure 10a). Then, from hypg5 to hypg9, the gene was suddenly not differentially expressed anymore, except for slight upregulation at hygp9 on the unspliced level. The gene then became greatly downregulated on the regular and spliced level at hypg15, but became even more upregulated on the unspliced level, up to a log fold change of 1.00 at hypg60, representing a doubling of transcript counts. The gene codes for ten different transcripts, four of them protein-coding, two nonsense-mediated decay transcripts, and four retained intron transcripts (Figure 10b). At the beginning protein-coding transcripts were responsible for around 99% of all transcript counts. The fraction of protein coding transcripts persistently dropped until hypg3, partially recovered between hypg5 and hypg9, decreased again at hypg15 and were partially restored again at hypg60. The amount of nonsense-mediated decay transcripts was constant for most of the time, until hypg15 and hypg60 where levels were increased. Additionally, for transcripts representing retained introns, an increase in transcript levels could be observed, however already starting at hypg75s. When comparing the effects on nonsense-mediated decay and retained intron transcripts, the kinetics looked very different from the overall effects on all transcripts, where nonsense-mediated decay progressively dropped in counts and retained introns had a steady upwards trend except for hypg5-hypg9 (Figure 8).

When looking in more detail at single transcripts, we identified that exons were distributed in an uneven manner: most exons were located at the end of the reversely transcribed gene, therefore between 102.08 Mbp and 102.09 Mbp on chromosome 14. There were two exons that were remote from the others, one at 102.102 Mbp, and one at 102.139 Mbp. Three of the ten transcripts used these exons, one protein-coding transcript exclusively (ENST00000558600). Upon looking at the count values for each transcript separately (Supplementary Figure S4), it became evident that this protein coding transcript was hardly transcribed in any condition. The other protein-coding transcript (ENST00000334701) that carried these remote exons had a baseline transcription of around 400 counts and did not change significantly for any comparison. The third, nonsense-mediated decay, transcript (ENST00000557234) had a baseline expression of around 10 counts and significantly increased for hypg15 and hypg60. The counts for protein-coding genes were mostly driven by ENST00000216281, for nonsense-mediated decay by ENST00000554401, and were distributed between all four transcripts for retained intron.

The different directions of change for unspliced and spliced transcripts could also be observed when directly plotting the sequencing coverage along the entire gene (Figure 10d). Here, the exons (1) and introns (2) of the exon-dense area was highlighted, which carried the majority of the overall reads (see total view in black box). We could identify the highest amount of exon reads for the control sample group and the lowest amount for hypg15. The amount of exon reads follows the order for protein-coding transcript counts from (b). In parallel, the amount of intronic sequences was slightly increased from shorter to longer hypergravity exposures with the highest amount identified for hypg60.

**Figure 11:**
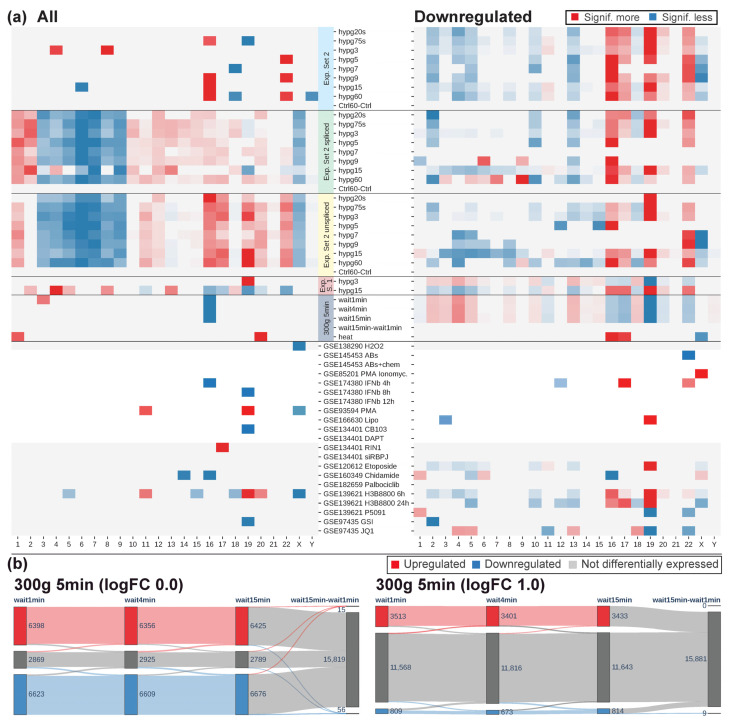
(**a**) Chromosomal distribution analysis of all data sets from Table 1. The same analysis as in Figure 7 was performed. If for a chromosome, the difference between expectation and real distribution was significant (FDR-adjusted *p* value of Fisher’s exact test below 0.05), this was indicated by a blue box if significantly less and a red box if significantly more. The stronger the color, the smaller the *p* value. (**b**) Temporal coherence of the centrifugation study, 5 min centrifugation at 300 *g* with different waiting times: 1 min (as quickly as possible), 4 min, and 15 min. The samples were compared to a control that was not centrifuged, except for a direct comparison between 15 min and 1 min waiting time after 5 min of centrifugation. The analysis was performed at an FDR-corrected *p* value cutoff of <0.05, and a log fold change cutoff of 0.0 and 1.0, respectively.

The observed centrifugation effects led to the question which influence centrifugation commonly used in laboratory work might have on transcriptomic analyses. We therefore exposed human Jurkat T cells to a hypergravity environment of 300× *g* for 5 min. We compared the data from Experiment Set 2 and the previously generated Experiment Set 1 to a centrifugation data set (300× *g* for 5 min) and to all external RNA-Seq data sets that were available on GEO on Jurkat T cells omitting gene knock out or knock down studies (Table 1, Figure 11). The Experiment Set 1 study had a similar layout as the Experiment Set 2 study, but only covered hypg3 (3 min) and hypg15 (15 min), and, most importantly, worked with poly-A RNA-Seq instead of rRNA depletion RNA-Seq, which does not cover transcripts that are not finished transcribing [40].

The centrifugation study applied 300× *g* for 5 min in a standard tabletop centrifuge. This is part of the standard protocol for RNA extraction for sequencing for suspension cell culture samples and is broadly used when splitting suspension cell cultures [35]. We wondered how our results on centrifugation at 9 *g* would transfer to standard cell culture practice. After the centrifuge stopped, different waiting times were applied to understand how quickly cells potentially affected by hypergravity return to their original state. This is especially important if samples in an assay have different waiting times, which could introduce bias. Therefore, there was a sample group without centrifugation, one with fixation after 1 min after centrifugation (the quickest possible time under standard lab conditions), one after 4 min, and one after 15 min. Additionally, we added a 42 °C heat shock (for 5 min) sample to apply another type of stress on the cells. All samples were analyzed by rRNA depletion RNA-Seq.

The external data sets contained data on oxidative stress by H_2_O_2_; activation of Jurkat immune cells by antibodies, chemokines, PMA + Ionomycin, Interferon IFNb, and simply PMA; reaction to cyclic DNA introduction via Lipofectamin; inhibition of parts of the NOTCH pathway, and inhibitors of different cellular functions, including splicing. Except for the PMA + Ionomycin and H_2_O_2_ treatment, all stimuli were applied for at least 2 h, therefore significantly longer than any of our internal time points. All data were generated from poly-A RNA-Seq. Comparing the number of differentially expressed genes, the data sets on 300× *g* 5 min centrifugation, 42 °C heat shock, PMA treatment, topoisomerase II inhibitor, NOTCH inhibitor, Palbociclib, splicing modulator, ubiquitin-specific protease inhibitor, and BRD4 inhibitor also had 1000s of genes differentially expressed. Interestingly, H_2_O_2_ treatment was skewed greatly towards upregulation, heat shock towards downregulation.

Next, the coherence of the data was tested compared to hypg20s and hypg60. This was performed in a similar way as in Figure 4. Here, it was calculated what percent of the upregulated/downregulated genes of one comparison were also upregulated/downregulated for another comparison. The actual value was divided by the predicted value; therefore, 1 is perfect random drawing; larger values than 1 indicate a strong overrepresentation and close to 0 a strong underrepresentation. Comparisons from Experiment Set 2 showed a strong coherence, both for upregulated and downregulated genes. Correlation to hypg20s was higher for early samples, to hypg60 for late samples. The spliced transcriptome data were well in line with the general set. Unspliced data were less in line with the general set, which could be driven by the fact that the regular fraction was mostly dominated by spliced transcripts. For Experiment Set 1, the first sample showed an inverted coherence compared to hypg20s and no coherence to hypg60. However, the second sample was in coherence with both, especially compared to hypg20s. This was especially interesting since Experiment Set 1 showed an inversion of differential gene expression between 3 and 15 min, which was not the case for Experiment Set 2. The centrifuge data set (300× *g* 5 min) showed strong inverted coherence behavior, compared to hypg20s, but only slightly compared to hypg60. Interestingly, for the heat shock comparison, upregulated genes were coherent with hypg60, but not with hypg20s or downregulated genes.

When regarding the external data sets, most were not or only in slight coherence with the Experiment Set 2 data. Transfection with plasmid DNA via Lipofectamine for 4 h caused very similar genes to be downregulated for hypg20s; however, only 88 genes were downregulated in total. The response to interferon beta caused an overlap of downregulated genes compared to hypg20s with a factor of 2.8.

Next, the radial distribution of differentially expressed genes was investigated (compare Figure 2). For comparison, the difference to the predicted radius was used. Experiment Set 1 showed a behavior in line with the Experiment Set 2 results for the late point in time and an inverted behavior for the early point in time: For Experiment Set 1 hypg3, upregulated genes located more in the center, downregulated genes at the periphery of the nucleus. Centrifugation at 300× *g* for 5 min also created an inverted pattern, similar to the Experiment Set 2 samples. Heat shock only showed a shift in the same direction as Experiment Set 2 for the upregulated genes, in line with its DEG coherence. For most external data sets, the average radius was not or only slightly shifted from expectation. The plasmid via Lipofectamine transfection was a prominent exception that deviated both for up- and downregulation. Additionally, IFNb treatment after 4 h had a shift of 0.46 µm of downregulated genes to the periphery, the strongest in the entire analysis.

Further, in accordance with the study encompassing Experiment Set 1 [40], the association of expression patterns with stress granules and P bodies was investigated (Supplementary Table S1). Experiment Set 2 samples and centrifugation (300× *g* 5 min) samples had a similar pattern, which none of the external data sets displayed.

Next, the chromosomal distribution analysis from Figure 7 was conducted on all data (Figure 11a). Chromosomes with significant differences in total DEG number were sparse, except for the Experiment Set 2 unspliced/spliced data. Skew in up- versus downregulation was more prominent for most data sets. Here, Experiment Set 2 showed a conserved behavior for the regular/spliced/unspliced transcriptome. For Experiment Set 1, the early comparison was inverted, and the late was in line with Experiment Set 2. The centrifugation (300× *g* for 5 min) data were also inverted in their chromosomal distribution. For the external data sets, only the two H3B8800 comparisons shared patterns with Experiment Set 2.

The centrifugation (300× *g* for 5 min) data were compared to the other stress data sets. After 5 min hypergravity, the comparisons had around 15,000 differentially expressed genes, by far the largest number compared to all data (Table 1). Widely accepted stress triggers, such as heat shock or oxidative stress, only caused around 5000 resp. 1000 genes to be expressed differentially. When assessing if waiting after centrifugation would cause a recovery towards the initial state, hardly any differences were noticed between 1 min, 4 min, and 15 min (Figure 11b). A direct comparison between 15 min and 1 min wait yielded only 15 upregulated and 56 downregulated genes. Depending on the aim of an analysis, higher fold change requirements might be applied to call a gene differentially expressed. When looking at genes that had at least double or half the counts (logFC cutoff of +/−1.0), there were still between around 3400 and 3500 upregulated and between around 700 and 800 genes downregulated. Only nine genes were downregulated in the direct comparison 15 min versus 1 min wait. None of them classified as protein-coding (not displayed in the figure). A waiting time of 15 min after centrifugation therefore still left the cell heavily impacted.

## 3. Discussion

In our study, we found a highly dynamic and mostly biphasic time course of gene expression response to hypergravity, which is not surprising due to the fact that gene expression networks are highly non-linear systems [47]. We were nevertheless able to characterize the dynamics in more detail and detected a “transition point” in the biphasic time course of differential gene expression at approximately 7 min, after which gene expression response showed no major changes up to 1 h hypergravity. Not only the number of differentially expressed genes was affected over time but also the strength of the differential expression.

We also found that upregulated genes were shifted towards the center of the nuclei, whereby downregulated genes were shifted towards the periphery. Interestingly, the “transition point” of gene movement correlated with differential gene expression, as downregulated genes moved inwards in the first 7 min before moving again towards the periphery. Upregulated differentially expressed gene expression was mostly located on chr16-chr22 in the first phase of cellular response to hypergravity. Protein-coding transcripts formed the majority with more than 90% of all differentially expressed genes and followed a continuous trend of downregulation, whereas retained introns demonstrated a biphasic time-course. Thus, the differential expression pattern analyzed in this study supports our hypothesis that Jurkat T cells respond to altered gravity via direct mechano-genetical coupling of gravitational forces to the nucleus, where genes are differentially expressed, partially depending on their nuclear location [39]. Experiments were performed with a T cell line to allow for cross-validation and comparability with our previous studies [36,39,40]. Although chromatin dynamic effects due to gravity changes in primary T lymphocytes have not yet been investigated, some studies suggest that chromatin modifications can also be expected in primary lymphocytes: In previous studies, we detected an overall downregulation of histone H3 acetylation in primary human T lymphocytes after the hypergravity phase of an suborbital ballistic rocket experiment [48], whereas a recent study identified modification of histone H3 lysine 27 trimethylation (H3K27me3) at TCRβ locus in murine thymocytes after chronic hypergravity exposure of 2× *g* for 21 days [49].

Importantly, the gene expression pattern of hypergravity response was not comparable with other stress factors such as oxidative stress, heat shock or inflammatory stress. This finding suggests that the transcriptional response to hypergravity is mediated by different signaling pathways and molecular processes. Nevertheless, an overlap with stress signaling pathways is for example HSP90 gene expression. Hsp90 is a transcriptional regulator by alteration of steady-state levels of transcription factors, modulation epigenetic modifiers and participation in the eviction of histones from the promoter regions [50]. In the immune system, Hsp90 is crucial for regulation of LAT expression in activated T cells [51], associated with T cell activation [52] and activation of tumor-specific immune responses [53,54].

While the application of cell culture techniques still represents the essential cornerstone of bioscientific experimental research [55], their methodological foundations and requirements are often not considered in practical applications. Thus, according to various studies and estimates, between 18% and 36% of cell lines are contaminated or misidentified, and additionally cell lines are often used beyond safe passage numbers, where cells no longer maintain key gene functions and consistent morphology [56]. In addition, there are insufficiently controlled influencing factors such as environmental instability in cell culture media [57], failure to monitor cell culture environments [58], and general deficiencies in methodological reporting in scientific publications [59]. Even in areas where there is clearer and unambiguous evidence of methodological issues, such as contaminated or misidentified cell lines, these have found little practical application over many years: for example, more than 30,000 studies with the effect of more than half a million citations have come from the use of well-established misidentified cell lines [60]. Cell culture supplements such as HEPES can also have consequences for cellular function and transcription [61]. Many studies and recommendations address this requirement of high standards in “good cell culture practice”, which is fundamental to the entire experimental cell biology [57,62]. In addition to the largely already known methodological challenges, basic research repeatedly leads to the identification of new—and previously only poorly known—influencing factors that should be taken into account in experimental standardization. For example, the influence of circadian rhythms is often not taken into account in cell culture and experimental protocols, although circadian transcriptional oscillations are detectable in almost all cells, especially in cells of the immune system [63,64].

The standardization and control procedures required for flight experiments in Space Life Sciences already contributed to the detection of significant effects in our earlier studies, which were caused by the cell culture environment in the flight hardware [30,31,33,36,39], and in later studies even by the handling of the cells [33]. Therefore, it is obvious to also investigate the effect of centrifugation, which is common routine in cell culture protocols. Direct cellular effects of hypergravity are well known for a broad range of cell types [14,15,16,17,18,19,20,21,22,23,24,25,26,27,28,30,31] and can be induced by even short-term and weak hypergravity (such as 1.8 g for 20 s) [30,31,33]. Routine cell culture protocols apply significantly higher and longer hypergravity exposures, e.g., 5 min at 300× *g* for cell harvesting for RNA isolation [35] and 5 min at 150 *g* for cell culture subculturing [34].

In our study, we applied this standard condition of 5 min 300 *g* on Jurkat T cells and detected a huge impact on the transcriptome compared to non-centrifuged samples with approximately 15,000 differentially expressed genes. If using a strict fold change cutoff of factor 2, more than 4000 genes were still affected. Waiting times of up to 15 min after centrifugation did not lead to any detectable response to return to baseline. Comparison with other stressors showed that the transcriptional response induced by routine centrifuge-based cell harvesting is stronger than oxidative stress induced by H_2_O_2_, inflammatory cell activation or heat stress. It must be assumed that cell centrifugation used as a routine procedure is, in principle, capable of subjecting the processed living cells to severe stress and leading to a massively altered transcriptome response.

The fact that the possible influence of hypergravity generated by centrifugation techniques on living cells remained undiscovered so far, might also be due to the fact that *GAPDH*, the typical “housekeeping” gene in quantitative PCRs (qRT-PCR) and commonly used at that time to normalize batch-to-batch variations in relation to target gene expression under investigation, belongs to exactly those genes whose expression is extremely robustly stable under altered gravity conditions [39,65,66].

Uncontrolled cell culture conditions can have a huge impact on the validity of results. For example, tumor cell lines in culture are subject to high clonal selection, which is highly sensitive to cell culture conditions [67]. Influencing factors that are not taken into account or controlled have a huge impact on the validity of conclusions drawn from experimental studies, where these aim to approximate physiological or pathophysiological conditions as closely as possible in order to make preclinical conclusions about human diseases and their treatment.

Since we have only tested one cell type in this study, no general conclusions can or should be drawn. Thus, we cannot conclude that all gene expression analyses obtained by previous routine methods reflect a “hypergravity world” and not conditions on Earth. Nevertheless, it must be recommended to carefully study the response of any cell types used for any experiments regarding the hypergravity time and levels applied during cell culture work. This is the only way to avoid, or at least quantify and control, any undesirable influence of hypergravity on the biological system generated by cell culture methods. It is possible that sensitivity as well as adaptive potential and adaptation speed of different cell types differ considerably. Therefore, we recommend internal validation of the centrifugation methods used, with respect to the experimental system and the target and measurement parameters. Since it can be assumed that experimental control groups will be exposed to the identical hypergravity loads as the experiment groups, in the best case, masking of the experimental effect may occur equally in experimental and control groups. In the worst case, different adaptation times after centrifugation, possibly caused by sample handling, or statistical chance alone due to the oscillation of the target parameters in the thermodynamically unstable system cell, lead to false results. Methodically, not only centrifugation but also other procedures such as microfiltration technologies and laminar wash technologies are available for separating cells from the liquid medium. Of course, the cellular influences of these technologies would also have to be carefully characterized.

In this study, the Jurkat T lymphocyte model was used to investigate gene expression response to hypergravity environments acting on human cells either at the systemic (e.g., military aviation or spaceflight), cellular (e.g., acceleration in the blood stream) or experimental level (e.g., cell culture centrifugation). Here, we characterized a rapid, strong, and dynamic biphasic response, that is stronger than previously studied stressors such as inflammation, heat shock, and oxidative stress. We hypothesize that hypergravity induces rapid and strong cellular effects at the level of gene expression regulation, which are not only physiologically relevant but have also received little attention in basic experimental systems (such as centrifugation of cell cultures) so far. From a scientific perspective, it is recommended that gravitational changes always be considered in biological and experimental systems, even if we as acting subjects live and work under the constant presence of the Earth’s gravity [68].

## 4. Materials and Methods

### 4.1. Preparation of Biological Samples

Cell culture samples were prepared as described previously [40]. Jurkat T cells were adjusted to a concentration of 5 × 10^6^ cells/mL without centrifugation. Samples were transferred into sterile 1 mL serological pipettes and centrifuged using a custom-built 9 g pipette centrifuge (KEK, Bad Schmiedeberg, Germany). The centrifuge stop time was 1.08 s (0.97 s–1.21 s min/max), therefore corresponding to 0.03% (60 min time point) up to 5.4% (20 s time point) of the total centrifugation time. Immediately after centrifugation, samples were eluted into RLT buffer. Total RNA was extracted by QIAGEN RNeasy Mini-Kits (Hilden, Germany). Samples were library-prepped with a rRNA depletion library and sequenced in paired end mode, 2 × 100 bp, with minimum 35 million reads.

### 4.2. 300× g Centrifugation and Heat Shock

For the centrifugation study (300× *g* 5 min), 5 mL of 1 × 10^6^ cells/mL Jurkat T cells was used that had not been exposed to centrifugation for 48 h. Four control samples were directly fixed. Three times four samples were centrifuged at 300× *g* for 5 min and fixed after one minute after centrifuge stop (fastest that was realistically possible while keeping experimental standard), after four minutes and after 15 min. The centrifuge stop time was 8.34 s (7.95 s–8.67 s min/max), therefore corresponding to 2.7% (5 min time point) of the total centrifugation time. The four heat shock samples were inserted into a 42 °C water bath under constant rotation to ensure mixing. The temperature curve was previously recorded: After one minute, the liquid was at 40 °C, from then on five minutes of heat exposure was performed before addition of RLT buffer to immediately stop all reactions.

### 4.3. RNA-Seq Pipelines

Canonical RNA-Seq (“regular”), separate quantification of the spliced and the unspliced transcriptome, chromosomal DEG distribution, differential exon usage, transcripts quantification, and stress granules/P bodies analysis were performed as described previously [40].

### 4.4. Principal Component Analysis

DESeq2-normalized counts were processed by the surrogate vector analysis batch effect removal package SVAseq [69]. The analysis identified three surrogate vectors based on sample group identity. These mostly represented sequencing depth, multimapper percentage, deduplication rate, GC content and intron percentage. After removal, the corrected counts were plotted as a PCA plot. This algorithm is supervised, therefore is aware of sample groups and will cluster them. However, the relation of sample groups to each other is not known to the algorithm, and therefore represents possible biological relations.

### 4.5. 3D mapping of Differentially Expressed Genes

Hi-C data from the previous mission 4th Swiss Parabolic Flight Campaign were generated and processed as previously described [39]. A mega map Hi-C map was generated based on all 12 samples. TADs were called with Juicer tools Arrowhead, version 2.20 ([70]. An external data track was used to define LADs, GSE94971. All data were processed as described in the Chrom3D tutorial [42]. 5000 representations of the nucleus with random seed were produced. The plausibility of the generated structures was assessed by clustering chromosomes based on the distance of their centers and angular distances, and by analyzing the average radial distance of chr18 and chr19 (Supplementary Figure S1). The small chromosomes chr16-chr22 clustered together, as expected from our previous work [39]. Additionally, chr18 was located more at the periphery of the nucleus than chr19, as expected from FISH studies on Jurkat T cells [71]. For analysis of gene distribution, all genes were mapped to their associated beads in the models. Expected radial distribution of DEGs was calculated by randomly drawing genes and labelling them as differentially expressed.

### 4.6. Time Course Analysis

Time course analysis was performed with the help of the package TCseq [45]. The number of clustering groups was varied between 2 and 12, 4 was chosen as the best compromise between simplicity and representing a large variety of time courses with at least 100 member genes. Genes that had an FDR-corrected *p* value of below 0.05 for their time courses were included in the analysis.

### 4.7. Coherence Analysis

Coherence between two comparisons defines how likely upregulated genes stay upregulated or downregulated genes stay downregulated. For the upregulated/downregulated genes of two comparisons, the smaller one was chosen. The number of genes that was upregulated/downregulated in the larger one and in the smaller one in parallel was divided by the number of genes in the smaller one (actual). Next the expectation value under the null hypothesis was calculated: If two comparisons were not coherent at all, the actual ratio should be equal to the number of genes in the larger upregulated/downregulated set divided by the total number of genes (expected ratio). Then, the actual ratio was divided by the expected ratio. Under the null hypothesis, this should be 1. If the value is close to 0 this indicates strongly inverted coherence; if it is much larger than 1, this indicates strong coherence.

## Figures and Tables

**Table 1:** Comparison of Experiment Set 2 to other experiments from our group and to all available Jurkat T Cell transcriptome data sets from GEO that are not transfections or knock downs. The utilized data sets were the regular, spliced, and unspliced transcriptome of the Experiment Set 2 data (rRNA depletion sequencing); the previous Experiment Set 1 study with 9 g for 3 min and 15 min (polyA sequencing); the internal centrifugation study with centrifugation at 300× *g* for 5 min in a standard benchtop centrifuge with different waiting times before RNA isolation after centrifugation, and a 5 min 42 °C heat stress data set; and external GEO data sets with accession number and treatment description. Application period of effects is given. Number of differentially expressed genes, both upregulated and downregulated, is given, based on a false discovery rate-adjusted *p* value below 0.05 and no fold change cutoff. Number of up- and downregulated genes is also visualized by bar charts, red for upregulated and blue for downregulated genes. Next, the correlation with hypg20s and hypg60 from Experiment Set 2 regular transcriptome is given, based on how many more of the significantly upregulated (up) or downregulated (down) genes of the smaller group were contained in the larger group, than expected. 1 indicates perfect match with the null hypothesis, that any overlaps between upregulated genes or downregulated genes could be explained by random drawing, much smaller than 1 indicates that much less genes were regulated continuously than expected (color-coded in violet), much larger indicates much more (color-coded in cyan). The columns’ 3D distribution indicates how many µm the average distribution of upregulated/downregulated genes deviated from expectation, based on the methodology from Figure 2. Deviation towards the nuclear center are indicated in green, deviation towards the periphery in yellow.

	Sample Name	Treatment	Effect Time	# of Differential Genes			Correlation—Up		Correlation—Down		3D Distribution	
				Up	Down	Total	hypg20s	hypg60	hypg20s	hypg60	Up	Down
**Exp. Set 2**	hypg20s	Centrifugation 9× *g*	20 s	962	753	1715		3.0		2.7	−0.29	0.48
	hypg75s	Centrifugation 9× *g*	75 s	2096	1830	3926	6.0	3.8	6.0	3.8	−0.33	0.40
	hypg3	Centrifugation 9× *g*	3 min	3001	3442	6443	3.9	3.1	3.4	3.4	−0.34	0.34
	hypg5	Centrifugation 9× *g*	5 min	896	535	1431	6.7	4.4	5.7	4.2	−0.31	0.24
	hypg7	Centrifugation 9× *g*	7 min	1021	1018	2039	4.8	4.6	2.3	4.6	−0.26	0.02
	hypg9	Centrifugation 9× *g*	9 min	1470	1164	2634	3.9	4.6	3.6	4.8	−0.27	0.16
	hypg15	Centrifugation 9× *g*	15 min	4191	4537	8728	2.2	3.2	2.6	3.4	−0.35	0.32
	hypg60	Centrifugation 9× *g*	60 min	3704	3295	6999	3.0		2.7		−0.27	0.21
	Ctrl60-Ctrl	Centrifugation 9× *g*	(60 min)	4	7	11						
**Exp. Set 2 spliced**	hypg20s	Centrifugation 9× *g*	20 s	373	253	626	12.6	3.6	15.5	2.6	−0.36	0.27
	hypg75s	Centrifugation 9× *g*	75 s	657	1753	2410	8.6	4.1	4.2	3.1	−0.43	0.15
	hypg3	Centrifugation 9× *g*	3 min	963	2767	3730	6.8	3.8	3.1	2.7	−0.45	0.17
	hypg5	Centrifugation 9× *g*	5 min	243	943	1186	8.2	4.3	3.5	3.1	−0.39	0.01
	hypg7	Centrifugation 9× *g*	7 min	290	1983	2273	6.7	4.1	1.5	2.7	−0.36	−0.17
	hypg9	Centrifugation 9× *g*	9 min	500	2541	3041	4.2	3.7	2.0	2.8	−0.34	−0.09
	hypg15	Centrifugation 9× *g*	15 min	1323	6353	7676	2.7	3.4	2.0	2.4	−0.47	0.22
	hypg60	Centrifugation 9× *g*	60 min	1855	5012	6867	3.7	4.7	1.7	2.8	−0.33	−0.06
	Ctrl60-Ctrl	Centrifugation 9× *g*	(60 min)	1	7	8						
**Exp. Set 2 unspliced**	hypg20s	Centrifugation 9× *g*	20 s	252	60	312	3.4	2.1			−0.42	0.06
	hypg75s	Centrifugation 9× *g*	75 s	2440	286	2726	2.4	2.1	3.3	2.5	−0.47	0.18
	hypg3	Centrifugation 9× *g*	3 min	3028	766	3794	2.1	1.8	2.5	2.2	−0.47	0.12
	hypg5	Centrifugation 9× *g*	5 min	1629	93	1722	2.5	2.1		2.2	−0.39	0.07
	hypg7	Centrifugation 9× *g*	7 min	2314	222	2536	2.2	2.0	0.9	1.4	−0.34	−0.06
	hypg9	Centrifugation 9× *g*	9 min	3599	294	3893	2.1	2.0	1.0	1.4	−0.37	−0.04
	hypg15	Centrifugation 9× *g*	15 min	5282	1580	6862	1.9	1.8	2.4	2.1	−0.43	0.40
	hypg60	Centrifugation 9× *g*	60 min	5911	1147	7058	1.9	2.1	1.9	2.3	−0.35	0.07
	Ctrl60-Ctrl	Centrifugation 9× *g*	(60 min)	0	0	0						
**Exp. Set 1**	hypg3	Centrifugation 9× *g*	3 min	2464	2593	5057	0.6	1.2	0.1	1.2	0.20	−0.27
	hypg15	Centrifugation 9× *g*	15 min	1844	3230	5074	3.3	2.3	3.0	2.0	−0.30	0.42
**300 *g* 5 min**	wait 1 min	Centrifugation 300× *g*	5 min + 1 min	8043	7030	15,073	0.2	0.8	0.2	1.4	0.28	−0.27
	wait 4 min	Centrifugation 300× *g*	5 min + 4 min	7906	7020	14,926	0.2	0.8	0.2	1.4	0.27	−0.26
	wait 15 min	Centrifugation 300× *g*	5 min + 15 min	8100	7130	15,230	0.2	0.8	0.2	1.4	0.27	−0.25
	wait 15 min–wait 1 min	Centrifugation 300× *g*	5 min + 15 min	15	56	71						0.01
	heat	42 °C	5 min	1279	3719	4998	0.7	3.5	1.1	2.0	−0.21	0.00
**External GEO reference data sets**	GSE138290	2 mM H_2_O_2_	30 min	1023	46	1069	0.7	1.3			−0.02	
	GSE145453	anti CD3/CD28 antibodies	2 h	1081	663	1744	1.7	1.0	0.6	0.8	−0.03	−0.16
	GSE145453	+100 ng/mL chemokines CXCL12 & CCL19	2 h	981	506	1487	2.1	1.2	0.9	1.0	−0.08	−0.07
	GSE85201	activation with 50 mM PMA + 1 µM Ionomycin	5 min	405	379	784	1.0	1.7	0.5	1.1	−0.02	−0.14
	GSE174380	100 IU/mL Interferon IFNb	4 h	246	391	637	0.8	1.1	2.8	1.5	−0.04	0.46
	GSE174380	100 IU/mL Interferon IFNb	8 h	359	98	457	0.7	0.9	1.0	1.2	0.05	−0.01
	GSE174380	100 IU/mL Interferon IFNb	12 h	347	204	551	0.9	1.3	1.7	1.8	0.02	−0.01
	GSE93594	20 ng/mL phorbol ester PMA	48 h	5840	5779	11,619	1.3	1.3	1.3	1.9	−0.03	−0.03
	GSE166630	pEF-GFP transfection with Lipofectamine LTX	24 h	68	88	156	1.1	0.7	4.8	1.4	−0.36	0.60
	GSE134401	NOTCH inhibition with CB-103	8 h	571	397	968	1.2	1.0	1.3	1.5	0.03	−0.02
	GSE134401	NOTCH inhibition with DAPT	8 h	17	29	46						
	GSE134401	NOTCH inhibition with RIN1	8 h	796	644	1440	0.9	1.7	1.9	1.4	−0.17	0.12
	GSE134401	NOTCH inhibition with siRBPJ	48 h	0	3	3						
	GSE120612	10 µM topoisomerase II inhibitor etoposide	4 h	5090	4656	9746	1.4	1.7	1.3	1.5	−0.08	0.08
	GSE160349	2 µM NOTCH inhibitor Chidamide	48 h	5802	4785	10,587	1.3	1.0	1.1	1.9	0.00	−0.05
	GSE182659	1 µM CDK4/6 inhibitor Palbociclib	72 h	2512	2476	4988	1.1	1.0	1.0	1.3	−0.07	−0.02
	GSE139621	30 nM splicing modulator H3B8800	6 h	7173	6674	13,847	1.7	1.5	1.3	1.4	−0.13	0.05
	GSE139621	30 nM splicing modulator H3B8800	24 h	4878	5054	9932	1.8	1.5	1.3	1.7	−0.08	0.02
	GSE139621	10 µM Ubiquitin-spec. prot. 7 inhibitor P5091	24 h	6356	5606	11,962	1.1	0.8	0.8	0.9	0.04	−0.10
	GSE97435	1 µM NOTCH inhibitor GSI	24 h	347	351	698	1.8	1.1	1.3	1.7	0.03	0.03
	GSE97435	500 nM BRD4 histone acetylase inhibitor JQ1	24 h	4291	5145	9436	1.0	1.0	0.7	1.2	0.07	−0.11

## Data Availability

The data sets generated and analyzed during the current study are available in the GEO (Gene Expression Omnibus) repository (www.ncbi.nlm.nih.gov/projects/geo), accession no. GSE221579.

## Supplementary Materials

The supporting information can be downloaded at: https://www.mdpi.com/article/10.3390/ijms24021351/s1.

## Abbreviations

DEGs	differentially expressed genes
DEU	differential exon usage
FDR	false discovery rate
GEO	Gene Expression Omnibus
Gx	Acceleration force (compared to Earth’s gravity) in x direction
Gz	Acceleration force (compared to Earth’s gravity) in z direction
Hi-C	high-throughput chromatin conformation capture
LADs	lamina associated domains
PMA	Phorbol-12-myristat-13-acetat
TADs	topology associated domains
