# Supplementary material for: Transcriptional Response in Human Jurkat T Lymphocytes to a near Physiological Hypergravity Environment and to One Common in Routine Cell Culture Protocols
Source: Int J Mol Sci. 2023 Jan 10;24(2):1351. doi: 10.3390/ijms24021351 (PMC9863927; doi:10.3390/ijms24021351)
Supplement: Supplementary file 1 [file ijms-24-01351-s001.zip › ijms-2161894-SI.pdf]

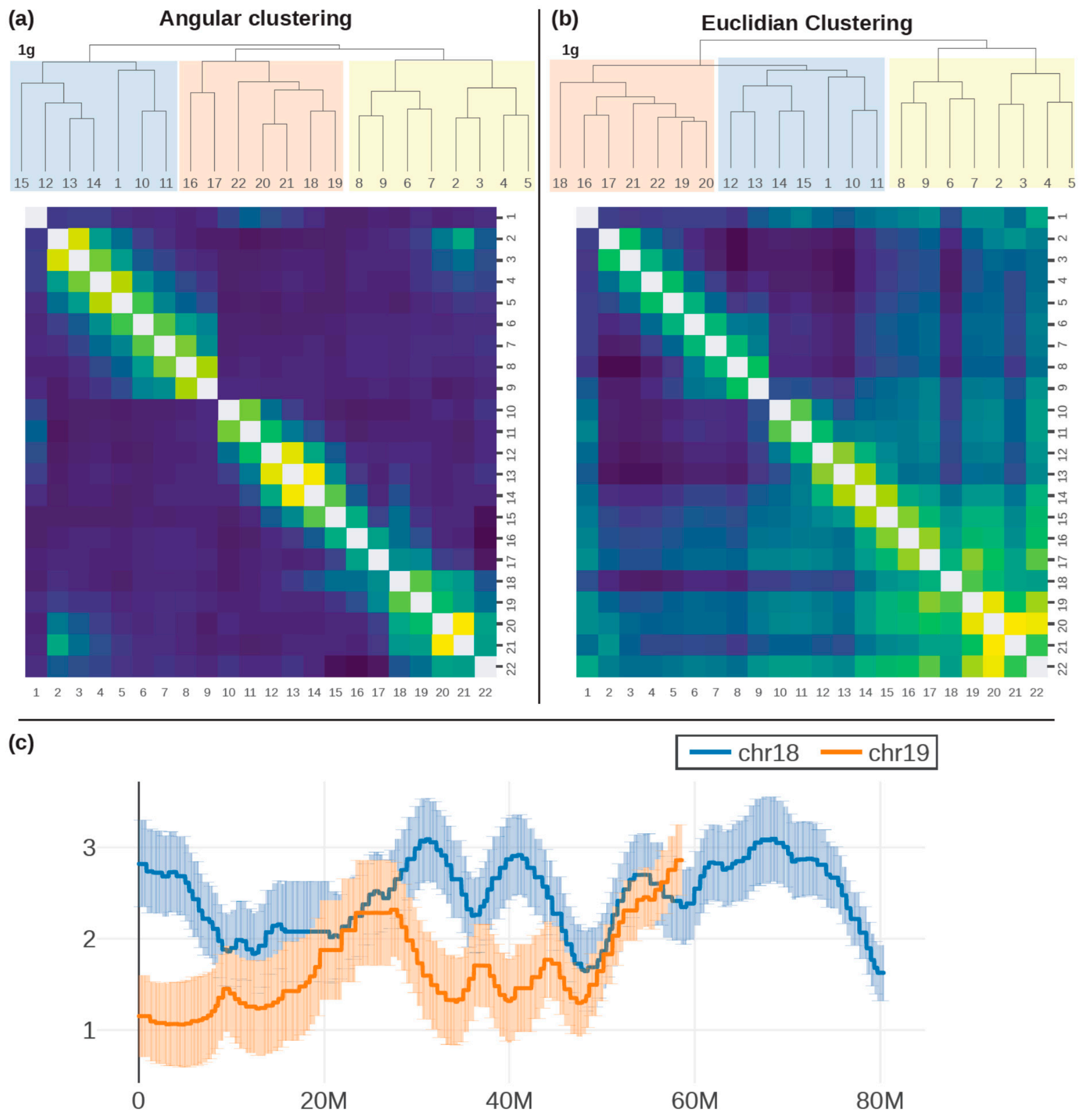

Supplementary Figure S1. Validation of 3d models from Figure 2. (a-b) The average location of chromosomes for all models was compared to each other. It is known from previous studies that chr16-chr22 behave very similar in altered gravity studies. It was controlled if they behave similarly when comparing the angular distance (as seen from the center of the nucleus) (a) and the Euclidean distance (length of the shortest connection between two point) (b) of chromosomes to each other. This is displayed as a clustering dendrogram and as a heatmap that indicates if two chromosomes have similar distances to other chromosomes (indicated in yellow, indicating proximity) or if they behave very differently (in blue, indicating remoteness). (c) The average radial distance of different parts of chromosomes 18 and 19. It is known from external studies that chr18 tends to localize more at the lamina of the nucleus and chr19 more in the center. It was controlled if the 3d models indicate such a predicted behavior.

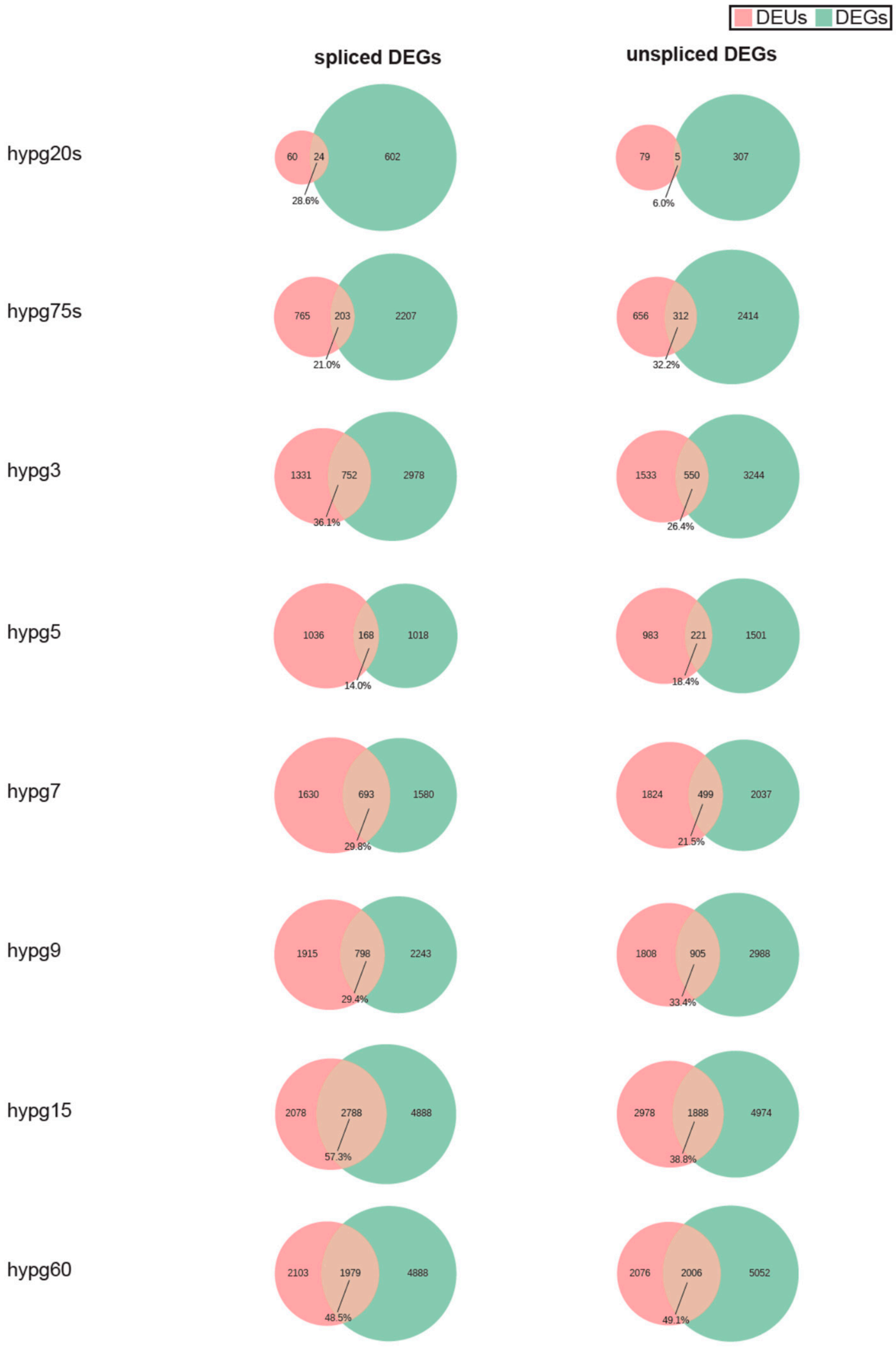

Supplementary Figure S2. Overlap between genes with differential exon usage (DEU, red) with genes that are differentially expressed (DEG, green). Fraction of overlapping DEU compared to all DEU is indicated in percent. Additional to Figure 9d.

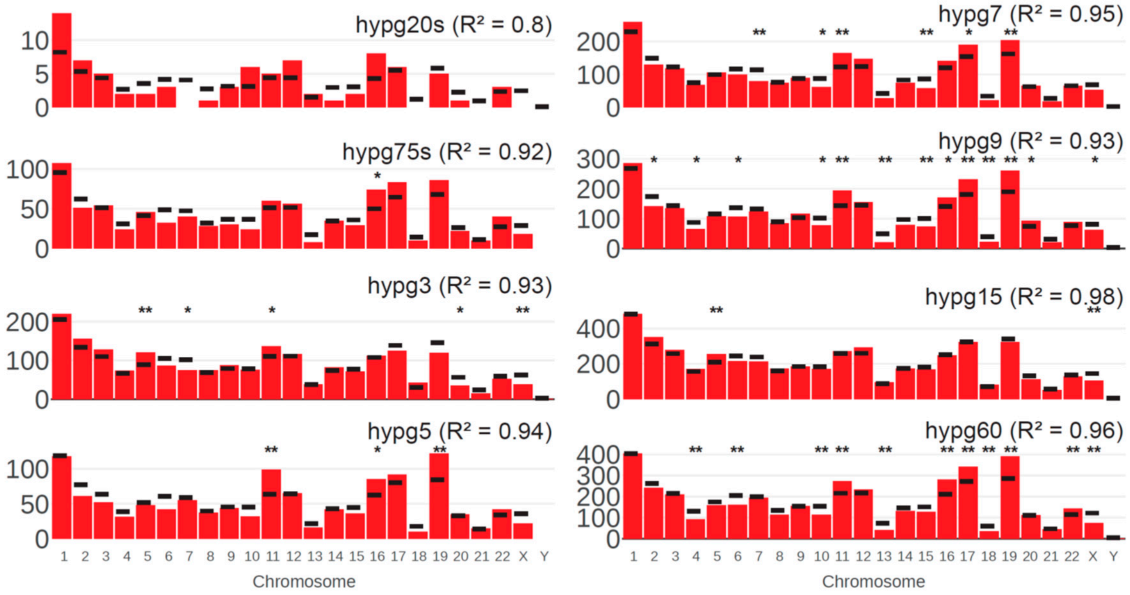

Supplementary Figure S3. Distribution of DEU genes along chromosomes. Genes from Figure 9, methodology as in Figure 7. Significant deviations from expectation are indicated by */** for a p-value below 0.05/0.01, based on a Fisher's exact test.

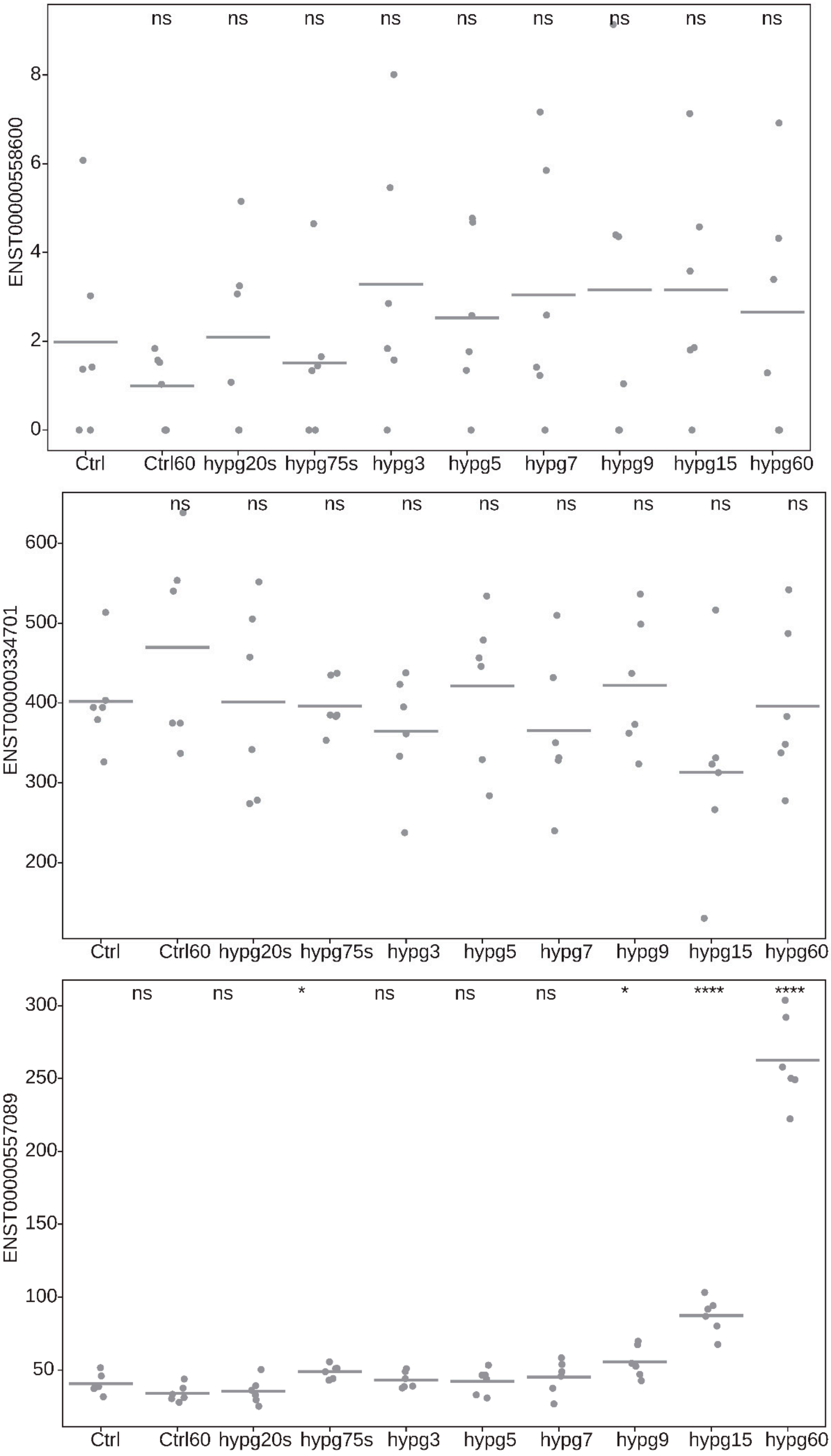

ENST00000558600
0
2
4
6
8
ns ns ns ns ns ns ns ns ns
Ctrl Ctrl60 hypg20s hypg75s hypg3 hypg5 hypg7 hypg9 hypg15 hypg60
ENST00000334701
200
300
400
500
600
ns ns ns ns ns ns ns ns ns
Ctrl Ctrl60 hypg20s hypg75s hypg3 hypg5 hypg7 hypg9 hypg15 hypg60
ENST00000557089
50
100
150
200
250
300
ns ns * ns ns ns * **** ****
Ctrl Ctrl60 hypg20s hypg75s hypg3 hypg5 hypg7 hypg9 hypg15 hypg60

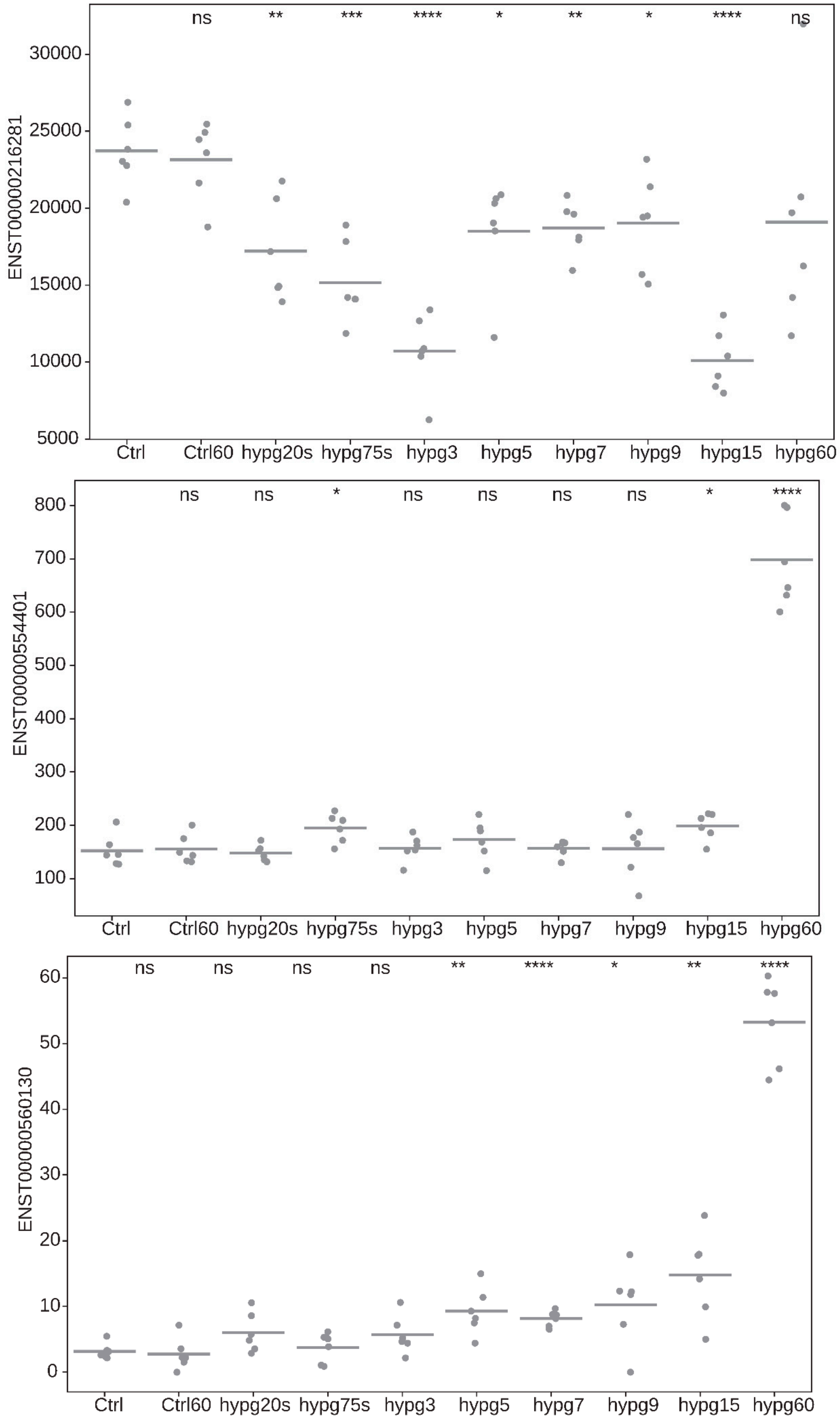

ns
**
***
****
*
**
*
****
ns
30000
25000
20000
15000
10000
5000
ENST00000216281
Ctrl
Ctrl60
hypg20s
hypg75s
hypg3
hypg5
hypg7
hypg9
hypg15
hypg60
ns
ns
*
ns
ns
ns
ns
*
****
800
700
600
500
400
300
200
100
ENST00000554401
Ctrl
Ctrl60
hypg20s
hypg75s
hypg3
hypg5
hypg7
hypg9
hypg15
hypg60
ns
ns
ns
ns
**
****
*
**
****
60
50
40
30
20
10
0
ENST00000560130
Ctrl
Ctrl60
hypg20s
hypg75s
hypg3
hypg5
hypg7
hypg9
hypg15
hypg60

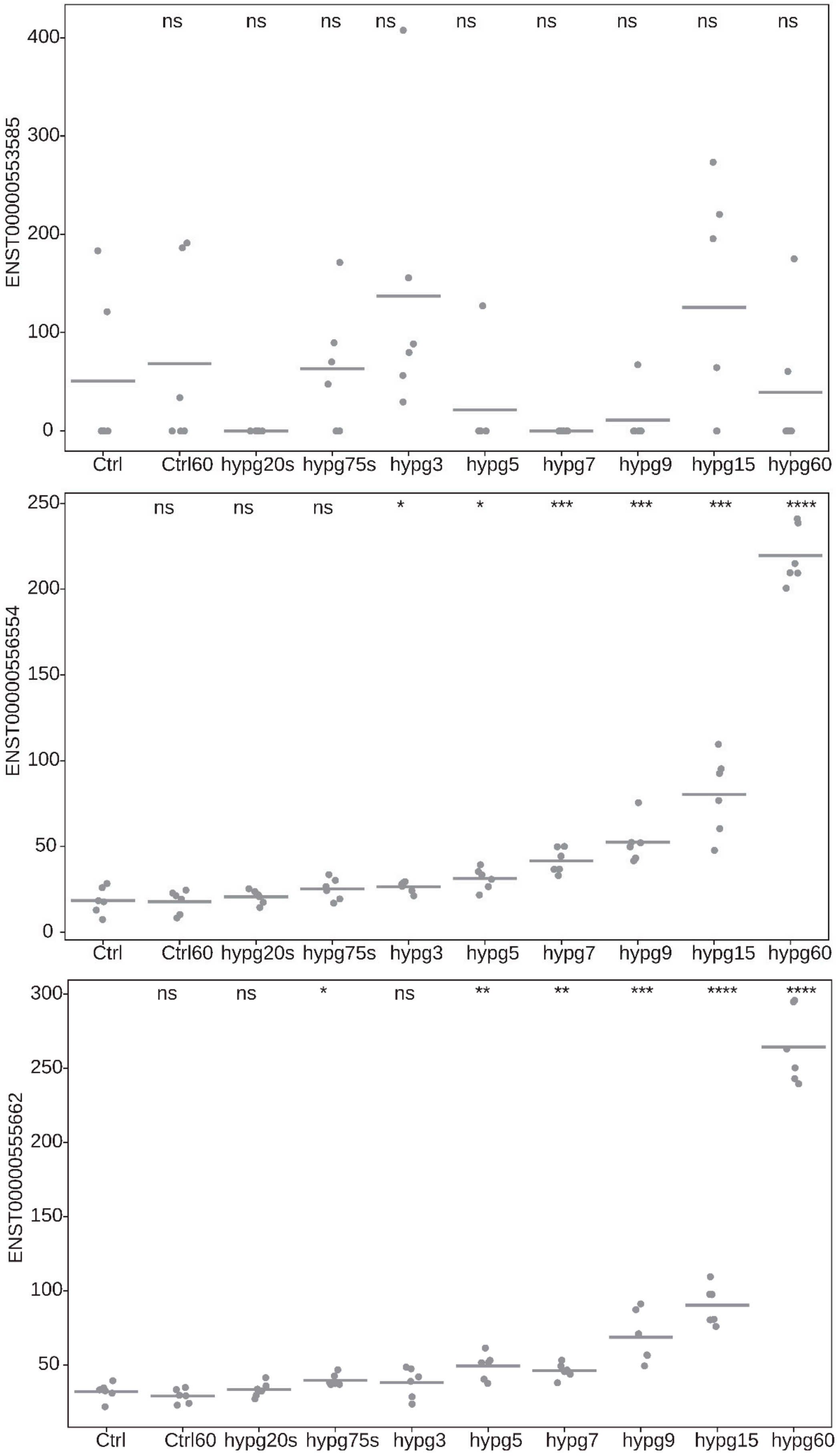
ENST00000553585
ENST00000556554
ENST00000555662
Ctrl
Ctrl60
hypg20s
hypg75s
hypg3
hypg5
hypg7
hypg9
hypg15
hypg60
ns
*
**
***
****

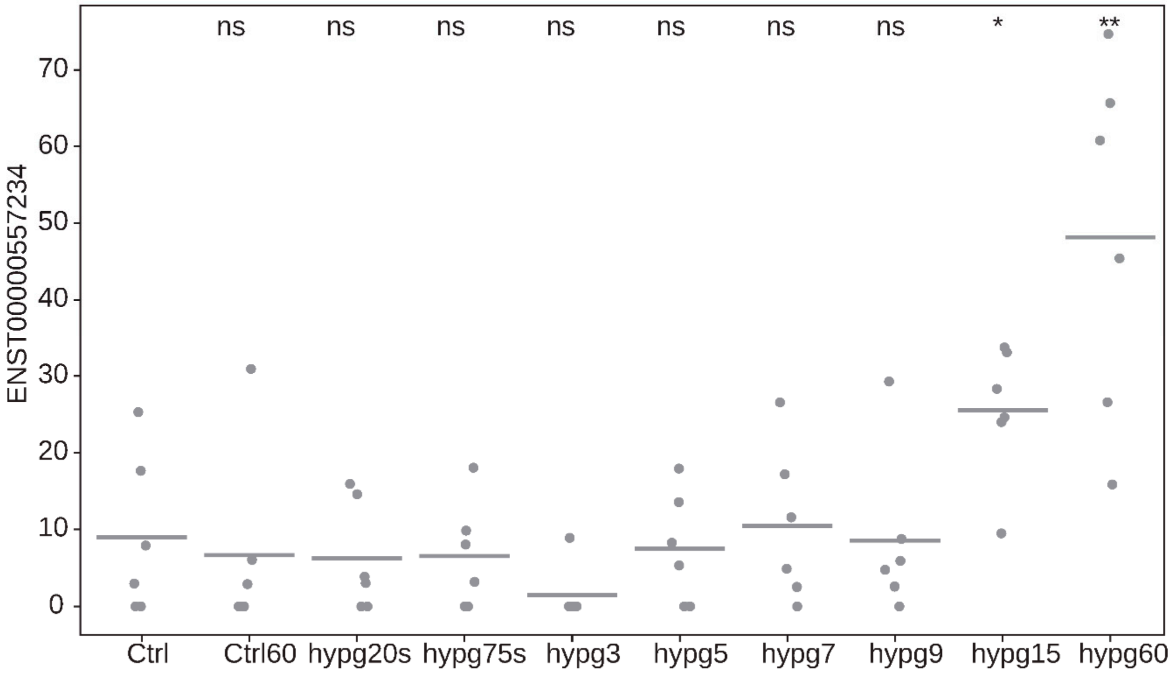

Supplementary Figure S4. Counts of all samples for each transcript of HSP90AA1, from Figure 10. Significant deviations from expectation are indicated by */**/***/**** for a p-value below 0.05/0.01/0.001/0.0001, based on a t test.

		Treatment	Effect time	up	down	total	SG - coding	P-Bodies	SG - Protein	SG - coding	P-Bodies	SG - Protein
Exp. Set 2	hypg20s		20s	962	753	1715	**		-**	-**		
	hypg75s		75s	2096	1830	3926	**		-**	-**	-**	**
	hypg3		3min	3001	3442	6443	**	-**	-**	-**	-**	**
	hypg5		5min	896	535	1431	**	**	-**	-**	-**	
	hypg7		7min	1021	1018	2039	**	**		-**	-**	**
	hypg9		9min	1470	1164	2634	**	**	-*	-**	-**	**
	hypg15		15min	4191	4537	8728		-**	-**		**	**
	hypg60		60min	3704	3295	6999	**		-**	-**	-**	**
	Ctrl60-Ctrl		(60min)	4	7	11						
Exp. Set 2 spliced	hypg20s		20s	373	253	626	**			-*		
	hypg75s		75s	657	1753	2410	**	**	-**	-**	-**	**
	hypg3		3min	963	2767	3730	**		-**	**	**	**
	hypg5		5min	243	943	1186	**	**	-*	-**	-**	**
	hypg7		7min	290	1983	2273	**	**		-**	-**	**
	hypg9		9min	500	2541	3041	**	**	-*	-**	-**	**
	hypg15		15min	1323	6353	7676		-**	-**	*	**	**
	hypg60		60min	1855	5012	6867	**	*	-**	-**	-**	**
	Ctrl60-Ctrl		(60min)	1	7	8						
Exp. Set 2 unspliced	hypg20s		20s	252	60	312	-**	-**	-**			
	hypg75s		75s	2440	286	2726		-**	-**	-**		
	hypg3		3min	3028	766	3794	-**	-**	-**	-**		
	hypg5		5min	1629	93	1722	*	-**	-**			
	hypg7		7min	2314	222	2536		-**		-**	-**	
	hypg9		9min	3599	294	3893		-**	-*	-**	-**	
	hypg15		15min	5282	1580	6862	-**	-**	-**	-**	-**	
	hypg60		60min	5911	1147	7058	-**	-**	-**	-**		
	Ctrl60-Ctrl		(60min)	0	0	0						
Exp. Set 1 2020	hypg3		3min	2464	2593	5057	**	**		-**	-**	-*
	hypg15		15min	1844	3230	5074	**	-**		*	**	
300g 5min	instant		5min	8043	7030	15073	**	**		-**	-**	
	wait1min		5min+1min	7906	7020	14926	**	**		-**	-**	
	wait15min		5min+15min	8100	7130	15230	**	**		-**	-**	
	wait15min-instant		5min+15min	15	56	71						
	heat	42°C	5min	1279	3719	4998	-**	-**	-**	**	**	**
External GEO reference data sets	GSE138290	2mM H2O2	30min	1023	46	1069	-*		-*			
	GSE145453	anti CD3/CD28 antibodies	2h	1081	663	1744				**		-**
	GSE145453	+ 100ng/ml chemokines CXCL12 & CCL19	2h	981	506	1487						-**
	GSE85201	activation with 50 mM PMA + 1µM Ionomycin	5min	405	379	784	-**	-**	-**	-**	-**	-**
	GSE174380	100IU/ml Interferon IFNb	4h	246	391	637	-**	**		*		
	GSE174380	100IU/ml Interferon IFNb	8h	359	98	457			**	**		*
	GSE174380	100IU/ml Interferon IFNb	12h	347	204	551	-*		*			**
	GSE93594	20ng/ml phorbol ester PMA	48h	5840	5779	11619			-**		**	**
	GSE166630	pEF-GFP transfection with Lipofectamine LTX	24h	68	88	156	-*	-**				
	GSE134401	NOTCH inhibition with CB-103	8h	571	397	968					-**	
	GSE134401	NOTCH inhibition with DAPT	8h	17	29	46				-*		
	GSE134401	NOTCH inhibition with RIN1	8h	796	644	1440	-**	-**				*
	GSE134401	NOTCH inhibition with siRBPJ	48h	0	3	3						
	GSE120612	10µM topoisomerase II inhibitor etoposide	4h	5090	4656	9746	**	**	-**	**	**	**
	GSE160349	2uM NOTCH inhibitor Chidamide	48h	5802	4785	10587	**		-**		*	**
	GSE182659	1uM CDK4/6 inhibitor Palbociclib	72h	2512	2476	4988	-**	**	*	-**	-**	-**
	GSE139621	30 nM splicing modulator H3B8800	6h	7173	6674	13847		*	**	**	*	
	GSE139621	30 nM splicing modulator H3B8800	24h	4878	5054	9932	*	-**	-**		*	
	GSE139621	10µM Ubiquitin-spec. prot. 7 inhibitor P5091	24h	6356	5606	11962	**	**	**	-**	-**	-**
	GSE97435	1µM NOTCH inhibitor GSI	24h	347	351	698	*			-**	-**	
	GSE97435	500nM BRD4 histone acetylase inhibitor JQ1	24h	4291	5145	9436	**	**	**	-**	-**	-**

Supplementary Table S1. Extension to Table 1. For all analyzed data sets, the significantly upregulated and downregulated genes were compared to the RNA composition of stress granules and P bodies, and the protein composition of stress granules. If significantly more (**/*: FDR-adjusted P value < 0.01/0.05) or less (-**/-*: FDR-adjusted P value < 0.01/0.05) differential genes were also contained in the stress granules / P bodies data set, this was indicated. This analysis was separately performed for upregulated and for downregulated genes.
